# Deliberate Practice, Functional Performance and Psychological Characteristics in Young Basketball Players: A Bayesian Multilevel Analysis

**DOI:** 10.3390/ijerph17114078

**Published:** 2020-06-08

**Authors:** Ahlan B. Lima, Juarez V. Nascimento, Thiago J. Leonardi, André L. Soares, Roberto R. Paes, Carlos E. Gonçalves, Humberto M. Carvalho

**Affiliations:** 1 Department of Physical Education, School of Sports, Federal University of Santa Catarina, Florianópolis, Santa Catarina SC 88040-900, Brazil; allanbenezar@gmail.com (A.B.L.); juarez.nascimento@ufsc.br (J.V.N.); asoares_fef012@hotmail.com (A.L.S.); 2 School of Physical Education, Physiotherapy and Dance, Federal University of Rio Grande do Sul, Porto Alegre, Rio Grande do Sul 90690-200, Brazil; thiago.leonardi@ufrgs.br; 3 Faculty Physical Education, University of Campinas, Campinas, São Paulo 13083-851, Brazil; robertopaes@fef.unicamp.br; 4 Faculty Sport Sciences and Physical Education, University of Coimbra, 3040-156 Coimbra, Portugal; cedgoncalves@gmail.com

**Keywords:** youth sports, maturation, motivation, talent development, specialization

## Abstract

Background: Early sport specialization has increased its popularity mostly based on the deliberate practice theory premises. In this study, we examined the influence of the age of onset of deliberate basketball practice on body size, functional performance (countermovement jump, line drill and yo-yo intermittent recovery level 1), motivation for achievement and competitiveness, motivation for deliberate practice and sources of enjoyment among young Brazilian basketball players. In addition, we adjusted for the influence of gender, age group, maturity status and state basketball federation on the outcomes. Methods: The sample included 120 female and 201 male adolescent basketball players aged 14.0 (1.7) years, on average. We grouped players by the age of onset of deliberate basketball practice as related to biologic maturation milestones (pre-puberty deliberate practice onset, mid-puberty deliberate practice onset and late-puberty deliberate practice onset). Results: There was no substantial variation among contrasting players by the onset of deliberate practice in all of the outcomes. Adjusting for gender, male players with late-puberty deliberate practice onset had better functional performance than players with pre- and mid-puberty onset of practice. Females players with late-puberty deliberate practice onset had slightly worst functional performance than players with pre- and mid-puberty onset of practice. Conclusions: Early deliberate basketball practice does not appear to provide an advantage for the development of physiological functions. Likewise, enjoyment, motivation for deliberate practice and motivation for achievement and competition do not appear to be negatively influenced by early deliberate basketball practice. The debate about the relationship between time spent in deliberate practice and performance development in young athletes will need to emphasize the coaching pedagogical quality and the training environment and account for informal practice and deliberate play.

## 1. Introduction

In the context of talent development, early sport specialization is often assumed as a premise for expertise attainment [1,2,3]. Early specialization popularity has increased mostly due to the influence of the deliberate practice theory [1,4,5]. The conceptual framework postulates that the accumulated hours of training through the sports career imply extensive deliberate practice starting in childhood followed by continuous expansion in the specialization years [6]. Promoters of early specialization often argue that early exposure to deliberate practice in a single sport will accelerate the development of athletes’ performance and skill level [3], and it is imperative to develop expertise in adulthood [1,7,8].

However, there has been extensive criticism of early specialization. Early specialization is associated with high rates of sport-related injury, lack of motivation for participation, and increased sports withdrawal (burnout) [8,9,10,11,12,13,14,15,16,17,18,19]. Data available is more abundant addressing the impact of early specialization on musculoskeletal structures [9,13,14,15,20,21]. When considering physiological function development or psychological determinants links with early specialization the available data are limited [8,11,12,22,23], at best. On the other hand, youth sports specialization research requires interdisciplinary approaches [23], as well as adjustments for confounding individuals (e.g., age, body size or gender) and contextual characteristics (e.g., sport context or socioeconomic environment) [11,23].

Similar to other youth sports contexts, the International Basketball Federation (FIBA) and its affiliated World Association of Basketball Coaches advise coaches and youth basketball programs to promote the engagement and commitment to basketball practice in supervised contexts as early as 5 years of age [24]. In basketball, body dimensions and specific functional capacities are important determinants of performance [25]. Furthermore, basketball-specific functional capacities appear to have a substantial influence on the selection/promotion process [26]. The emphasis—or probably overemphasis—on body size and functional capacities likely contributes to an overrepresentation of early maturing players at early age groups [26,27]. Hence, maturity-associated variation on body dimensions, functions, and behavioral characteristics should be considered in the interpretation of young players’ performance and development.

On the other hand, in this study, we focus on two main psychological factors associated with deliberate practice and expertise attainment in sport [28,29,30]. The first outcome of interest is motivation, in particular achievement and competitiveness motivation and deliberate practice motivation. The athlete’s motivation has been highlighted as a relevant factor to distinguish those who progress through talent development programs [30,31,32]. In particular, achievement motivation reflects the willingness to work hard and perform well, be challenged by difficult tasks, express high internal standards of excellence, and a desire to win in a competition against others [29]. Furthermore, motivation relates to the athlete’s engagement and willingness to be exposed to deliberate practice [29,30], translated in the will to become an excellent performer (excel), and the will to improve in a competition. The second factor of interest is sport enjoyment. Enjoyment in youth sport has been noted to be a primary factor for initiating and maintain committed to long term involvement in sport [33,34,35]. Enjoyment may be defined as pleasure, liking and fun for the sports practice [36]. A negative impact of early specialization on enjoyment has been observed in young athletes [37].

There is a lack of consensus in the literature to define specialization [11]. It may be conceptualized as year-round participation in a single “signature” sport, with limited participation in potential sport alternatives, and with a deliberate focus on training and development in the pursuit of elite status [11,12,38,39]. Also, youth sports participation and specialization can be conceptualized as a continuum. However, there are no clear references to define early specialization or late specialization. A solution is to interpret specialization relative to pubertal growth [5]. Assuming the influence of deliberate practice framework on youth sports specialization, we may consider the start of a “signature” sport deliberate practice as related to biological maturation milestones that describe the pubertal growth period [i.e., the age of initiation of the pubertal growth spurt and the age at peak height velocity (PHV)]. Hence, we may consider the original operationalization of deliberate practice that defines it as any training activity undertaken with the specific purpose of increasing performance, requiring cognitive and/or physical effort and relevant to promoting positive skill development [4]. The biological maturation milestones can be defined with available knowledge in the literature [40] and we can use Bayesian multilevel modeling to perform a meta-analysis to derive the estimates [41]. Then players may be labeled as the pre-puberty onset of deliberate practice (i.e., before age of onset of pubertal growth), the mid-puberty onset of deliberate practice (i.e., between the onset of pubertal growth and age at PHV) or late-puberty onset of deliberate practice (i.e., after the age of PHV).

Given the interdisciplinary nature of young players´ development [32,42,43], modeling approaches need to account for the potential influences of individual and contextual characteristics, as well as different levels and sources of variation on the outcomes [26,44]. In youth sports surveys, samples are often non-representative and imbalanced, with noisy data that can potentially lead to biased and unreliable inferences. As previously noted [44,45], the Bayesian multilevel modeling approach offers a flexible approach that naturally considers the hierarchical data structure. Bayesian methods allow us to combine the information known before seeing the data (i.e., the prior uncertainty concerning a parameter or hypothesis expressed as a probability distribution) with what is learned from the observed data (i.e., the likelihood of the data conditioned on the parameter or hypothesis) to update our knowledge expressed as the posterior distribution [46]. Hence, Bayesian methods provide a natural approach to account for different sources of inferential uncertainty [47].

In the present study, we examined the influence of the age starting basketball deliberate practice on functional performance and psychological characteristics in youth basketball. To allow a comprehensive interpretation, we illustrate the use of Bayesian multilevel modeling to estimate the variation in the outcomes accounting for cross-classified nesting, i.e., within and between variation by gender, competitive age group, estimated maturity status and state basketball federation (given the contrasts in organizational structures in Brazilian youth basketball across states).

## 2. Materials and Methods

### 2.1. Data

In this study, we considered data from surveys with cross-sectional data collected from 2015 to 2019 in youth basketball. The total sample comprised observations in 321 Brazilian adolescent basketball players aged 14.0 (1.7) years, on average, with a range between 9.5 and 17.9 years. The sample included 120 female and 201 male players. Players were engaged in clubs and competition from three state federations: São Paulo, Santa Catarina and Rio Grande do Sul. All federations are affiliated in the Confederação Brasileira de Basketball (Brazilian Basketball Confederation). Furthermore, all state federations have female and male youth basketball competitions each year. The São Paulo basketball federation (from the southeast part of Brazil) is the most representative in Brazil, as it has most of the teams in the highest professional championship. The Santa Catarina and the Rio Grande do Sul basketball federations have established traditions in youth basketball and organize a yearly state-level adult championship. Data were collected at each basketball club facility. All players participated in regular training sessions (3–5 sessions; 270–450 min per week) with their clubs. Clubs participated in a competitive season, which in Brazil typically runs between February/March until November/December comprising about 20–30 games per season. Given the variation between state federations for competitive age groups, we grouped players as under-11, under-13, under-15 and under-17, assuming a two-year range, which is the most common competitive age group in Brazilian youth sports. Distribution of players by gender and age group across the basketball federations is presented as supplementary material.

We obtained ethical approval from the authors´ institutional ethics committee. The participants were informed about the nature of the survey, that participation was voluntary and that they could withdraw from the study at any time. All participants and their parents or legal guardians provided written informed consent.

### 2.2. Chronological Age and Anthropometry

We calculated chronological age by subtracting birth date from the day of testing to the nearest 0.1 year. Stature was measured with a portable stadiometer (Seca model 206, Hanover, MD, USA) to the nearest 0.1 cm. Body mass was measured with a calibrated portable balance (Seca model 770, Hanover, MD, USA) to the nearest 0.1 kg. Reliability estimates for the observer are published elsewhere [32].

### 2.3. Maturity Status

Maturity status was determined based on the gender-specific maturity offset protocol [48]. The offset equations predict time before or after PHV based on chronological age and stature. Based on maturity offset, the participants, ranging from −2.99 to +5.42 years from/to PHV, were grouped into three maturity status categories for analysis: pre-PHV (PHV ≤ −1.00 year; *n* = 33), circa-PHV (−1.00 < PHV < +1.00 year; *n* = 103) and post-PHV (PHV ≥ + 1.00; *n* = 185). The range of distance to PHV to classify players by maturity status in the present study was larger and conservative than previous studies using age at PHV [49], assuming the limitations of the offset equation [48,50].

### 2.4. Basketball Deliberate Practice

The age of deliberate practice onset in basketball was considered as the self-reported age when athletes started formal training and competition in basketball, under the supervision of a coach within a youth basketball program registered in the state basketball federation, and with no participation in practice and competition in other organized sport. However, deliberate play [1] and informal participation in other sports, previous or after the onset of deliberate basketball practice was not considered in this study. Hence, we assume the limits of our data to describe the continuum of sport participation of the sample and caution are advised to interpret the data. We considered two biologic maturation milestones, the age of onset of the pubertal growth spurt and the age at PHV to interpret age starting basketball deliberate practice. The biologic milestones references were estimated considering data from longitudinal growth studies [40]. We estimated the gender-specific age of pubertal growth spurt onset using Bayesian multilevel modeling to perform a meta-analysis [41]. Similarly, we set the gender-specific references for the age at PHV based on a meta-analysis of 25 and 10 studies for female and male adolescents, respectively [40]. The reference age of the pubertal growth spurt onset was 9.4 (95% Credible Interval, CI 9.0 to 9.8) years and 11.1 (95% CI 10.8 to 11.5) years for girls and boys, respectively. The reference age at PHV was 11.9 (95% CI 11.8 to 12.0) years and 13.9 (95% CI 13.8 to 14.0) years for girls and boys, respectively. We grouped the players by the onset of deliberate basketball practice as follows: pre-puberty deliberate basketball practice onset (n = 156), the players who started practice before the reference age of pubertal growth spurt onset; mid-puberty deliberate basketball practice onset (n = 125), the players starting practice between the reference ages of pubertal growth spurt onset age and at PHV; late-puberty deliberate basketball practice onset (n = 40), the players starting practice after the reference age at PHV.

### 2.5. Functional Performance

We used the vertical jump with countermovement [51], a short-term maximal running protocol, the line drill test [52,53] and intermittent endurance test, the yo-yo intermittent recovery level 1 test (yo-yo IR1) [54] to examine functional performance. The tests were performed in two sessions separated by at least 48 h. The first session included the vertical jump and line drill test, and the second session the yo-yo IR1. A standardized warm-up was taken by all athletes before testing. Details about the functional performance procedures and reliability estimates are available elsewhere [32,43,55].

### 2.6. Psychological Measures

The psychological factors were assessed using the deliberate practice motivation questionnaire [28], the work and family orientation questionnaire [56] and the Sources of Enjoyment in Youth Sports [35]. The deliberate practice motivation questionnaire was originally designed for chess [28,29]. The questionnaire is composed of 18 items, rated similarly on a 5-point Likert scale, considering two dimensions of deliberate practice: will to compete and will to excel. We used an adapted version for basketball, translated and validated to Portuguese [30]. The adapted version to basketball included both long term goals (“I want to be a professional Basketball player”), and basketball-specific changing situations (“I like tough drills in practice because they help me to improve my skills” or “I prefer to play 3 on 3 with friends rather than practicing hard”) [30]. The reliability of the adapted Portuguese version has been reported with data in youth basketball from the same age range of the present study elsewhere [30]. The work and family orientation questionnaire is composed of 19 items, rated on a 5-point Likert scale (1 = completely disagree to 5 = completely agree), assessing four dimensions of achievement: personal unconcern (lack of concern with others’ opinions), work (the desire to face challenging tasks the desire to practice and perform well), mastery (the desire to face challenging tasks) and competitiveness (the desire to be better when compared to others). For the present study, we only used the last three subscales in the present study, consistent with previous observations with similar samples of youth basketball [30,32,43]. The players from the São Paulo Basketball Federation completed a study-and-pencil questionnaire, while the remaining completed an online form of the questionnaires. All questionnaires were completed under the supervision of a researcher during the data collection at the clubs training facilities. We used the 28-item Portuguese version of the sources of enjoyment in youth sport questionnaire [57]. The questionnaire examines five dimensions: self-referenced competencies, others-referenced competencies, effort expenditure, affiliation with peers and positive parental involvement. Each questionnaire item is rated on a 5-point Likert scale (1 = completely disagree to 5 = completely agree). The questionnaire showed good reliability [57].

### 2.7. Data Analysis

We used Bayesian multilevel models to examine variation by the onset of deliberate basketball practice, gender, age group, maturity status and state basketball federation on adolescent Brazilian basketball players´ body dimensions, functional performance and psychological characteristics. For interpretative convenience and computational efficiency, we standardized the outcomes. Each player´s outcome was estimated as a function of his/her characteristics, i.e., onset of deliberate basketball practice, gender, age group, maturity status and state (for player i, with indexes j, k, l, m and n for the onset of deliberate basketball practice, gender, age group, maturity status, and state, respectively), and we allowed female and male players to vary when grouped by the onset of deliberate basketball practice:

(1) $$y_{i}=\beta^{0}+\alpha_{j\left[i\right]}^{basketball\;practice}+\alpha_{k\left[i\right]}^{gender}+\alpha_{l\left[i\right]}^{age\;group}+\alpha_{m\left[i\right]}^{maturity\;status}+\alpha_{n\left[i\right]}^{state}+\alpha_{j\left[i\right],\;k\left[i\right]}^{basketball\;practice.gender}$$

The terms after the intercept are modeled as group effects (also referred to as random effects) drawn from normal distributions with variances to be estimated from the data:

$$\alpha_{j\left[i\right]}^{basketball\;practice}\;\sim \;N\;\left(0,\;\sigma_{basketball\;practice}^{2}\right),\;\mathrm{for}\;j=1,\;2,\;3.$$

$$\alpha_{k\left[i\right]}^{gender}\;\sim \;N\;(0,\;\sigma_{gender}^{2}),\;\mathrm{for}\;k=1,\;2.$$

$$\alpha_{l\left[i\right]}^{age\;group}\;\sim \;N\;(0,\;\sigma_{age\;group}^{2}),\;\mathrm{for}\;l=1,\;2,\;3\;\mathrm{and}\;4.$$

$$\alpha_{m\left[i\right]}^{maturity\;status}\;\sim \;N\;(0,\;\sigma_{maturity\;status}^{2}),\;\mathrm{for}\;m=1,\;2,\;3.$$

$$\alpha_{n\left[i\right]}^{state}\;\sim \;N\;(0,\;\sigma_{state}^{2}),\;\mathrm{for}\;n=1,\;2,\;3.$$

$$\alpha_{j\left[i\right],\;k\left[i\right]\;}^{basketball\;practice.gender}\;\sim \;N\;(0,\;\sigma_{basketball\;practice,gender}^{2}),\;\mathrm{for}\;j=1,\;2,\;3\;\mathrm{and}\;k=1,\;2.$$

An important step of the Bayesian methods is the selection of priors. Given that human behavior and performance tends to be heterogenous, particularly in youth sports contexts, we were intentionally conservative with our interpretations. Hence, we used weakly informative priors to regularize our estimates. We used normal priors (0,1) for the population-level parameter (i.e., intercept) and the group-level parameters. By standardizing the outcomes and using a normal (0,1) prior for the parameters, we state that effects among group-level estimates are unlikely to be greater than one standard deviation of the outcome. We run four chains for 2000 iterations with a warm-up length of 1000 iterations for each model. The models were inspected and validated using posterior predictive checks [45]. The Bayesian estimations were implemented using R statistical language [58], with the “brms” package [59] which call Stan [60].

## 3. Results

Characteristics of the youth basketball players adjusted for gender are shown in Table 1. All but nine maturity offset values were positive for the female players. A total of 138 of maturity offset values were positive for male players. In general, most of the players in the present sample were beyond the age at PHV. The range of onset of deliberate basketball practice was between 5 and 17 years for female players, and between 5 and 16 for male players.

Given the extensive results of our modeling approach, the estimates and uncertainty (80% credible intervals) grouped by the onset of deliberate basketball practice for female and male young basketball players are shown in Table 2 and Table 3, respectively. Although the results are shown separately, the marginal estimates are based on joint models, as described previously, after back-transformation from the standardized model-based estimates. The standardized outcomes plotted by the onset of deliberate basketball practice adjusted for by gender are available as supplementary material. There was no substantial variation between players grouped by the onset of deliberate basketball practice for body size, functional performance, motivation for deliberate practice, motivation for achievement and competitiveness and sources of enjoyment (Supplementary Figures S1–S5). However, when adjusting for gender, female players with a late-puberty onset of deliberate basketball practice had slightly worst functional performance values than players with a pre- and mid-puberty onset of practice (Table 2). Male players with a late-puberty onset of deliberate basketball practice had better functional performance values than players with a pre- and mid-puberty onset of deliberate basketball practice (Table 3). As for the motivation for deliberate practice, motivation for achievement and competitiveness and sources of enjoyment, there was no variation between players grouped by the onset of deliberate basketball practice, both for female and male players.

The posterior predictions and uncertainty (80% and 50% credible intervals) plotted by age group, maturity status, gender and state basketball federation are also presented as supplementary material. Overall, the present predictions are consistent with our previous reports accounting for the age- and maturity-related variation on functional performance and psychological characteristics [26,43,61]. In addition, there was considerable variation by gender for body dimensions, as expected (Supplementary Figure S8), for functional performance outcomes (Supplementary Figure S12). There was no apparent variation associated with the context of training and competition for body dimensions and functional performance. As for deliberate practice motivation, adolescent female players likely have slightly lower values for both will to excel and will to compete (Supplementary Figure S16). Furthermore, for achievement and competitiveness motivation adolescent female players likely have somewhat lower values for all dimensions than adolescent male players (Supplementary Figure S20). As for sources of enjoyment, scores appear to vary by gender only for others-referenced competencies (Supplementary Figure S24). The context of training and competition appeared to influence players´ psychological characteristics, in particular sources of enjoyment dimensions.

## 4. Discussion

In this study, our primary interest was to examine the influence of the onset of deliberate basketball practice on functional performance and psychological characteristics in youth basketball. Considering the need to adopt interdisciplinary approaches to interpret young players´ performance and development [26,42,43,62], we accounted for the confounding influence of players´ characteristics (gender, age and maturity status) and contextual characteristics (state basketball federations) on body dimensions, functional performance and psychological characteristics.

The delivery of youth sports programs in the last decades has changed substantially [38]. Youth sports programs often create a highly targeted, athlete-centered environment generally focused on early specialization. Youth sports programs often create a highly targeted, athlete-centered environment generally focused on early specialization [62]. Consequently, early specialization appears to be promoted as a mainstream path for talent development in team sports, as those who achieve professional status in adult sports may need to be engaged in youth sports academies at early pre-pubertal ages [63,64]. However, caution is warranted with this interpretation. Data have shown that to achieve a professional adult level, it may not be necessary—and even detrimental—to be included in talent development programs such as youth academies at a particularly young age [65]. Nevertheless, children and adolescents engaged in multi-sports developmental programs may have limited opportunities for later entry in talent development programs. This may be the case, at least, in youth sports that promote early engagement in their programs such as basketball.

Conditional on our data, there was no substantial variation on functional characteristics between players when group by the onset of deliberate basketball practice. Furthermore, players with a later onset of deliberate basketball practice appear to have slightly better functional performance, adjusting for age group, maturity status and state basketball federation. From a physiological performance perspective, early exposure to deliberate practice in basketball does not seem to accelerate the development of athletes’ performance, as argued by early specialization advocates [3,66]. Our data imply that there is no reason to narrow the opportunities for players with later onset of deliberate practice, likely late maturing boys in youth basketball. Inadvertently, by early targeting children and promoting early development and selection, youth basketball programs and coaches are selecting/promoting and reducing the pool of available players after pubertal growth to mostly early maturing players.

Our predictions suggest the need to be cautious—and to consider gender differences when interpreting players´ functional performance. Albeit the large uncertainty in our prediction, it was apparent that female players with a later onset of deliberate basketball practice had worst performance than their peers with an earlier onset of deliberate practice. Pubertal changes and sexual maturation in girls is accompanied by smaller gains in muscle mass, a widening of the hips relative to shoulders and an increase in body fat percentage [67]. Likely, earlier sports participation/practice may be important for a better adjustment of young female players´ athletic performance to pubertal changes. Hence, coaches, parents, youth basketball organizers and ultimately, athletes should be aware of the need to promote young girls’ participation in sports, either early specialization or sampling in different sports, not only to increase their chances to achieve expertise in basketball, but to promote girls a positive development through basketball practice [68].

Criticism of early specialization has been based mostly on the negative impacts the rates of sport-related injury, increased sports withdrawal and decreased motivation for participation, along with other health problems including burnout, overuse injuries and overtraining [9,10]. Conditional on the data, early onset of deliberate basketball practice had no negative influence on young players´ motivation and scores for sources of enjoyment, adjusting for individual and contextual factors. Furthermore, the scores in all motivation dimensions and sources of enjoyment scores were consistently high in both female and male athletes. Considering the deliberate practice framework, our observations appear to concur with athletes’ retrospective ratings of deliberate practice being perceived as enjoyable. These observations do not fit well with the original definition of deliberate practice for musicians [12]. Overall, our results lead us to agree with suggestions to shift the focus of the debate about specialization. Emphasis should be placed on the quality of sports instruction, regardless of the level of performance or accumulated hours of practice [3,69]. Conditional on the data, the training environments in Brazilian youth basketball programs appear to be sufficient to motivate players to be committed with deliberate practice. Young basketball players are likely willing to perform well and be challenged by difficult tasks and with a desire to compete. Furthermore, players seem to enjoy their training environments and deliberate practice. In these two relevant psychological outcomes, there was no apparent negative effect of early onset of deliberate basketball practice. These observations suggest that at least some of the negative consequences of early sport specialization may be avoided with appropriate coaching and sport skill instruction [3]. Hence, youth basketball programs and interested stakeholders should invest in the promotion of the quality of coaching, adequate training and competitive environment for the athletes.

The use of Bayesian multilevel models allowed us to explore the interacting influences of individual and contextual factors on functional capacities and psychological characteristics. The present results considering age- and maturity-related variation are consistent with our previous observations in female and male young basketball players [26,32,43]. Mainly, these results give us confidence in our models’ validity to replicate previous observations when combining and including more participants and other sources of variation. As noted previously, caution is warranted when interpreting maturity-associated variation on the outcomes, given the limitations of the maturity offset equations. On the other hand, several dimensions of motivation and enjoyment varied across the state basketball federations (see Supplementary material). These results highlight the need to consider the influence of the training environments [12]. In addition, it highlights a limitation in our analysis, which is the lack of information about coaching.

In general, youth sports specialization discussion is dichotomized as “early specialization” versus “early diversification” [12]. In our study, we used the onset of deliberate basketball practice as a left censor of the data. This indicator may be insufficiently sensitive to mark the beginning of the athletes´ specialization in basketball. Nevertheless, we assumed that our indicator marked year-round participation in a single sport (basketball), potentially limiting participation in potential sport alternatives. As stated in the methods section, information about players´ deliberate play and informal participation in other sports, previous or after the onset of deliberate basketball practice, was not retained in our surveys. Hence, we assume the limitations of our data and highlight the need for caution when generalizing our data and interpretations. Considering the influence of nature (genes) or nurture (environment) on players’ performance development, the decision about the start and type of engagement in specific-sport training programs (early specialization, sampling or late specialization) is probably the most significant in the hands of children/adolescent and their parents. Nevertheless, future research into the patterns of children and adolescents in youth sports should consider a multidimensional continuum that is reflected by several continuous variables including chronological age, the onset of deliberate practice, growth and maturation patterns, amount of main-sport coach-led practice, amount of main-sport youth-led play, amount of other-sports coach-led practice, amount of other-sports youth-led play and perhaps the number of sports in which an athlete engages [65,70].

## 5. Conclusions

Assuming an interdisciplinary perspective with the present sample of adolescent basketball players, there was no substantial variation on body size, functional characteristics and psychological characteristics between players when group by the onset of deliberate basketball interpreted as related to puberty. Hence, there was no apparent advantage of the early accumulation of deliberate basketball practice for the development of specific physiological functions. Likewise, there was no negative effect of early onset of deliberate basketball practice on enjoyment, motivation for deliberate practice or motivation for achievement and competition. Furthermore, all psychological scores were consistently high across the players in the present study. Based on our data and models, it was apparent the need to consider gender differences and the context of the youth basketball programs when interpreting the relevance of deliberate basketball practice and specialization. Given an appropriate environment and pedagogical approach, structured youth basketball may potentially provide a positive environment for players´ development and commitment to training and excellence attainment. Coaches should refine their pedagogical strategies, adjusting for the importance of the interactions among physical growth, biologic maturity status, and accumulation of deliberate practice and its influence on functional performance and psychological characteristics. Furthermore, researchers and coaches should consider the potential contributions of deliberate play and informal practice on players´ development. Hence, further interdisciplinary research about youth sports developmental paths is warranted. Overall, we concur with the need to focus the debate about specialization in youth sports and developmental paths on the pedagogical quality of the coach and the training environment [3].

## Figures and Tables

**Table 1:** Marginal estimates and 80% credible intervals of young Brazilian basketball players adjusted by gender.

	All Sample	Female	Male
	(*n* = 321)	(*n* = 120)	(*n* = 201)
Chronological age, years	14.1 (13.6 to 14.6)	14.2 (14.0 to 14.4)	13.9 (13.8 to 14.1)
Maturity offset, years	1.46 (0.44 to 2.46)	2.17 (1.99 to 2.35)	0.71 (0.57 to 0.84)
Onset of deliberate basketball practice, years	10.4 (9.6 to 11.1)	10.1 (9.8 to 10.4)	10.6 (10.4 to 10.9)
Stature, cm	168.1 (165.9 to 170.3)	165.0 (163.0 to 166.0)	171.0 (170.0 to 172.0)
Body mass, kg	63.2 (61.1 to 65.3)	60.5 (58.7 to 62.3)	65.9 (64.6 to 67.3)
Countermovement jump, cm	28.7 (26.8 to 30.6)	25.8 (25.1 to 26.4)	31.5 (31.0 to 32.1)
Line drill test, s	34.7 (33.7 to 35.7)	35.4 (35.1 to 35.7)	34.0 (33.7 to 34.3)
Yo-yo IR1, m	673 (391 to 956)	543 (497 to 590)	815 (781 to 851)
**Deliberate Practice Motivation**			
Will to excel, 1–5	4.09 (3.59 to 4.59)	3.88 (3.78 to 3.98)	4.27 (4.20 to 4.35)
Will to compete, 1–5	4.31 (3.96 to 4.63)	4.25 (4.18 to 4.33)	4.41 (4.36 to 4.47)
**Achievement and Competitiveness Motivation**			
Mastery, 1–5	4.14 (3.74 to 4.52)	4.06 (3.99 to 4.13)	4.27 (4.21 to 4.32)
Work, 1–5	4.36 (3.94 to 4.74)	4.27 (4.21 to 4.33)	4.54 (4.49 to 4.59)
Competitiveness, 1–5	3.76 (3.40 to 4.19)	3.58 (3.50 to 3.67)	3.84 (3.77 to 3.90)
**Sources of Enjoyments in Youth Sports**			
Self-referenced competencies, 1–5	4.41 (4.07 to 4.71)	4.38 (4.31 to 4.45)	4.53 (4.48 to 4.58)
Others-referenced competencies, 1–5	3.79 (3.43 to 4.19)	3.63 (3.51 to 3.74)	3.84 (3.76 to 3.92)
Effort expenditure, 1–5	4.69 (4.45 to 4.87)	4.73 (4.68 to 4.79)	4.72 (4.68 to 4.76)
Affiliation with peers, 1–5	4.45 (4.21 to 4.66)	4.46 (4.39 to 4.54)	4.48 (4.43 to 4.54)
Positive parental involvement, 1–5	4.35 (4.04 to 4.63)	4.45 (4.36 to 4.55)	4.35 (4.28 to 4.42)

**Table 2:** Marginal estimates and 80% credible intervals of female young Brazilian basketball players by the onset of deliberate basketball practice.

	Onset of Deliberate Basketball Practice		
	Early Starters	Starters during pubertal growth	Late starters
Stature, cm	153.3 (151.0 to 155.6)	153.2 (151.0 to 166.0)	153.6 (151.1 to 156.1)
Body mass, kg	55.0 (51.4 to 58.6)	54.2 (50.8 to 57.6)	57.6 (53.6 to 61.6)
Countermovement jump, cm	24.1 (22.0 to 26.2)	23.8 (21.8 to 25.8)	22.3 (20.0 to 24.6)
Line drill test, s	35.5 (34.5 to 36.4)	36.6 (35.7 to 37.5)	36.9 (35.9 to 37.8)
Yo-yo IR1, m	402.3 (274.2 to 529.8)	378.9 (261.8 to 497.4)	342.8 (208.0 to 478.2)
**Deliberate Practice Motivation**			
Will to excel, 1–5	3.89 (3.65 to 4.12)	3.77 (3.54 to 3.99)	3.89 (3.64 to 4.13)
Will to compete, 1–5	4.20 (4.03 to 4.36)	4.24 (4.08 to 4.40)	4.23 (4.05 to 4.40)
**Achievement and Competitiveness Motivation**			
Mastery, 1–5	4.07 (3.91 to 4.22)	4.04 (3.89 to 4.19)	4.04 (3.87 to 4.20)
Work, 1–5	4.23 (4.06 to 4.39)	4.26 (4.10 to 4.41)	4.27 (4.10 to 4.43)
Competitiveness, 1–5	3.53 (3.28 to 3.79)	3.43 (3.18 to 3.67)	3.43 (3.17 to 3.69)
**Sources of Enjoyments in Youth Sports**			
Self-referenced competencies, 1–5	4.30 (4.14 to 4.46)	4.33 (4.18 to 4.54)	4.37 (4.19 to 4.54)
Others-referenced competencies, 1–5	3.47 (3.17 to 4.77)	3.42 (3.13 to 3.70)	3.33 (3.01 to 3.64)
Effort expenditure, 1–5	4.69 (4.54 to 4.82)	4.72 (4.57 to 4.86)	4.70 (4.54 to 4.85)
Affiliation with peers, 1–5	4.60 (4.41 to 4.79)	4.59 (4.41 to 4.77)	4.48 (4.26 to 4.69)
Positive parental involvement, 1–5	4.53 (4.31 to 4.72)	4.51 (4.31 to 4.72)	4.39 (4.13 to 4.63)

**Table 3:** Marginal estimates and 80% credible intervals of male young Brazilian basketball players by the onset of deliberate basketball practice.

	Onset of Deliberate Basketball Practice		
	Early starters	Starters during pubertal growth	Late starters
Stature, cm	167.2 (165.3 to 169.2)	168.9 (166.9 to 171.1)	167.9 (165.4 to 170.4)
Body mass, kg	66.7 (63.6 to 69.8)	66.4 (63.2 to 69.7)	68.1 (64.1 to 72.1)
Countermovement jump, cm	30.6 (28.8 to 32.5)	30.6 (28.6 to 32.5)	32.6 (30.1 to 35.1)
Line drill test, s	34.6 (33.8 to 35.4)	34.4 (33.6 to 35.2)	33.7 (32.7 to 34.8)
Yo-yo IR1, m	687.3 (583.7 to 794.1)	772.5 (663.0 to 884.6)	1142.1 (989.8.0 to 1294.0)
**Deliberate Practice Motivation**			
Will to excel, 1–5	4.31 (4.11 to 4.52)	4.19 (3.97 to 4.42)	4.31 (4.07 to 4.56)
Will to compete, 1–5	4.32 (4.17 to 4.47)	4.37 (4.20 to 4.52)	4.35 (4.18 to 4.51)
**Achievement and Competitiveness Motivation**			
Mastery, 1–5	4.29 (4.15 to 4.42)	4.27 (4.12 to 4.41)	4.26 (4.10 to 4.41)
Work, 1–5	4.40 (4.26 to 4.55)	4.43 (4.28 to 4.58)	4.44 (4.28 to 4.60)
Competitiveness, 1–5	3.84 (3.62 to 4.07)	3.74 (3.50 to 3.97)	3.74 (3.48 to 3.99)
**Sources of Enjoyments in Youth Sports**			
Self-referenced competencies, 1–5	4.27 (4.12 to 4.42)	4.30 (4.15 to 4.45)	4.34 (4.17 to 4.52)
Others-referenced competencies, 1–5	3.94 (3.66 to 4.20)	3.88 (3.60 to 4.15)	3.79 (3.46 to 4.10)
Effort expenditure, 1–5	4.70 (4.56 to 4.83)	4.73 (4.60 to 4.87)	4.71 (4.56 to 4.86)
Affiliation with peers, 1–5	4.50 (4.34 to 4.67)	4.49 (4.31 to 4.66)	4.38 (4.16 to 4.59)
Positive parental involvement, 1–5	4.41 (4.22 to 4.60)	4.39 (4.20 to 4.59)	4.27 (4.01 to 4.51)

## Supplementary Materials

The following are available online at https://www.mdpi.com/1660-4601/17/11/4078/s1, Table S1. Distribution of observations by gender and age group across the basketball state federations, Table S2. Posterior estimations of youth basketball players by gender and age group, Figure S1. Posterior predictions for stature (a) and body mass (b) by onset of deliberate basketball practice in young female and male basketball players (80% and 50% credible intervals), Figure S2. Posterior predictions for countermovement jump (a), line drill test (b) and yo-yo intermittent recovery test level-1 (c) by onset of deliberate basketball practice in young female and male basketball players (80% and 50% credible intervals)., Figure S3. Posterior predictions for will to excel (a) and will to compete (b) by onset of deliberate basketball practice in young female and male basketball players (80% and 50% credible intervals), Figure S4. Posterior predictions for mastery (a), work (b) and competitiveness (c) by onset of deliberate basketball practice in young female and male basketball players (80% and 50% credible intervals), Figure S5. Posterior predictions for self-referenced competences (a), others-referenced competences (b), effort expenditure (c), positive parental involvement (d) and affiliation with peers (e) by onset of deliberate basketball practice in young female and male basketball players (80% and 50% credible intervals), Figure S6. Posterior predictions for body dimensions by age group in young female and male basketball players, controlling for maturity status, age starting basketball practice and state basketball federation (80% and 50% credible intervals), Figure S7. Posterior predictions for body dimensions by maturity status in young female and male basketball players, controlling for age group, age starting basketball practice and state basketball federation (80% and 50% credible intervals), Figure S8. Posterior predictions for body dimensions in young female and male basketball players, controlling for age group, maturity status, age starting basketball practice and state basketball federation (80% and 50% credible intervals), Figure S9. Posterior predictions for body dimensions by state basketball federation in young female and male basketball players, controlling for age group, maturity status and age starting basketball practice (80% and 50% credible intervals), Figure S10. Posterior predictions for functional capacities by age group in young female and male basketball players, controlling for maturity status, age starting basketball practice and state basketball federation (80% and 50% credible intervals), Figure S11. Posterior predictions for functional capacities by maturity status in young female and male basketball players, controlling for age group, age starting basketball practice and state basketball federation (80% and 50% credible intervals), Figure S12. Posterior predictions for functional capacities in young female and male basketball players, controlling for age group, maturity status, age starting basketball practice and state basketball federation (80% and 50% credible intervals), Figure S13. Posterior predictions for functional capacities by state basketball federation in young female and male basketball players, controlling for age group, maturity status and age starting basketball practice (80% and 50% credible intervals), Figure S14. Posterior predictions for deliberate practice motivation dimensions by age group in young female and male basketball players, controlling for maturity status, age starting basketball practice and state basketball federation (80% and 50% credible intervals), Figure S15. Posterior predictions for deliberate practice motivation dimensions by maturity status in young female and male basketball players, controlling for age group, age starting basketball practice and state basketball federation (80% and 50% credible intervals), Figure S16. Posterior predictions for deliberate practice motivation dimensions in young female and male basketball players, controlling for age group, maturity status, age starting basketball practice and state basketball federation (80% and 50% credible intervals), Figure S17. Posterior predictions for deliberate practice motivation dimensions by state basketball federation in young female and male basketball players, controlling for age group, maturity status and age starting basketball practice (80% and 50% credible intervals), Figure S18. Posterior predictions for achievement and competitiveness motivation dimensions by age group in young female and male basketball players, controlling for maturity status, age starting basketball practice and state basketball federation (80% and 50% credible intervals), Figure S19. Posterior predictions for achievement and competitiveness motivation dimensions by maturity status in young female and male basketball players, controlling for age group, age starting basketball practice and state basketball federation (80% and 50% credible intervals), Figure S20. Posterior predictions for achievement and competitiveness motivation dimensions in young female and male basketball players, controlling for age group, maturity status, age starting basketball practice and state basketball federation (80% and 50% credible intervals), Figure S21. Posterior predictions for achievement and competitiveness motivation dimensions by state basketball federation in young female and male basketball players, controlling for age group, maturity status and age starting basketball practice (80% and 50% credible intervals), Figure S22. Posterior predictions for sources of enjoyment dimensions by age group in young female and male basketball players, controlling for maturity status, age starting basketball practice and state basketball federation (80% and 50% credible intervals), Figure S23. Posterior predictions for sources of enjoyment dimensions by maturity status in young female and male basketball players, controlling for age group, age starting basketball practice and state basketball federation (80% and 50% credible intervals), Figure S24. Posterior predictions for sources of enjoyment dimensions in young female and male basketball players, controlling for age group, maturity status, age starting basketball practice and state basketball federation (80% and 50% credible intervals), Figure S25. Posterior predictions for sources of enjoyment dimensions by state basketball federation in young female and male basketball players, controlling for age group maturity status and age starting basketball practice (80% and 50% credible intervals).
