# Supplementary material for: Deliberate Practice, Functional Performance and Psychological Characteristics in Young Basketball Players: A Bayesian Multilevel Analysis
Source: Int J Environ Res Public Health. 2020 Jun 8;17(11):4078. doi: 10.3390/ijerph17114078 (PMC7312187; doi:10.3390/ijerph17114078)
Supplement: Supplementary file 1 [file ijerph-17-04078-s001.pdf]

Supplementary Material

Deliberate Practice, Functional Performance and Psychological Characteristics in Young Basketball Players: A Bayesian Multilevel Analysis

Ahlan B. Lima [1], Juarez V. Nascimento [1], Thiago J. Leonardi [2], André L. Soares [1], Roberto R. Paes [3], Carlos E. Gonçalves [4] and Humberto M. Carvalho [1,*]

[1] Department of Physical Education, School of Sports, Federal University of Santa Catarina, Florianópolis, SC 88040-900 Santa Catarina, Brazil; allanbenezar@gmail.com (A.B.L.); juarez.nascimento@ufsc.br (J.V.N.); asoares_fef012@hotmail.com (A.L.S.)

[2] School of Physical Education, Physiotherapy and Dance, Federal University of Rio Grande do Sul, Porto Alegre, 90690-200 Rio Grande do Sul, Brazil; thiago.leonardi@ufrgs.br

[3] Faculty Physical Education, University of Campinas, Campinas, 13083-851 São Paulo, Brazil; robertopaes@fef.unicamp.br

[4] Faculty Sport Sciences and Physical Education, University of Coimbra, 3040-156 Coimbra, Portugal; cedgoncalves@gmail.com

* Correspondence: hmoreiracarvalho@gmail.com; Tel.: +554-837-218-526

Received: 11 April 2020; Accepted: 29 May 2020; Published: date

Table 1. Distribution of observations by gender and age group across the basketball state federations.

	**Santa Catarina**		**São Paulo**		**Rio Grande do Sul**		**Total**
	Female	Male	Female	Male	Female	Male	
Under 11	1		11	23			35
Under 13	12		33	71		10	126
Under 15	17		25	60		14	116
Under 17	17		4	18		5	44
Total	47		73	172		29	321

Table S2. Posterior estimations of youth basketball players by gender and age group.

	Female				Male			
	under 11	under 13	under 15	under 17	under 11	under 13	under 15	under 17
Chronological age, years	11.3 (11.2 to 11.4)	13.1 (13.0 to 13.2)	15.0 (14.9 to 15.1)	16.9 (16.8 to 17.0)	11.2 (11.1 to 11.3)	13.0 (12.9 to 13.1)	14.9 (14.8 to 15.0)	16.8 (16.7 to 16.9)
Maturity offset, years	–0.42 (–0.57 to–0.26)	1.22 (1.13 to 1.32)	2.92 (2.82 to 3.01)	4.28 (4.14 to 4.41)	–1.73 (–1.87 to–1.59)	–0.09 (–0.17 to–0.01)	1.60 (1.52 to 1.69)	2.96 (2.83 to 3.10)
Starting basketball practice, years	9.1 (8.6 to 9.6)	9.6 (9.3 to 10.0)	10.4 (10.0 to 10.7)	11.1 (10.6 to 11.6)	9.7 (9.2 to 10.2)	10.3 (10.0 to 10.5)	11.0 (10.7 to 11.3)	11.7 (11.2 to 12.2)
Stature, cm	151.0 (149.0 to 153.0)	161.0 (160.0 to 162.0)	169.0 (168.0 to 171.0)	170.0 (168.0 to 172.0)	159.0 (156.0 to 161.0)	169.0 (168.0 to 170.0)	177.0 (176.0 to 178.0)	178.0 (176.0 to 180.0)
Body mass, kg	47.8 (44.6 to 50.8)	56.6 (54.6 to 58.5)	65.0 (62.9 to 66.9)	64.8 (62.2 to 67.4)	54.3 (51.4 to 57.2)	63.1 (61.4 to 64.8)	71.5 (69.8 to 73.2)	71.3 (68.9 to 73.8)
Countermovement jump, cm	21.6 (20.4 to 22.8)	24.1 (23.3 to 24.8)	27.0 (26.2 to 27.8)	28.9 (27.8 to 29.9)	27.7 (26.5 to 28.9)	30.2 (29.6 to 30.9)	33.2 (32.5 to 33.9)	35.0 (33.9 to 36.1)
Line drill test, s	38.9 (38.3 to 39.4)	36.2 (35.9 to 36.5)	34.4 (34.0 to 34.7)	33.8 (33.4 to 34.3)	37.3 (36.8 to 37.9)	34.7 (34.4 to 35.0)	32.9 (32.6 to 33.2)	32.3 (31.8 to 32.8)
Yo-yo IR1, m	267 (176 to 355)	472 (366 to 472)	642 (586 to 697)	765 (688 to 839)	554 (472 to 638)	707 (663 to 751)	930 (883 to 976)	1053 (978 to 1127)
**Deliberate Practice Motivation**								
Will to excel, 1–5	3.90 (3.76 to 4.06)	3.94 (3.82 to 4.06)	3.81 (3.69 to 3.93)	3.88 (3.74 to 4.01)	4.30 (4.16 to 4.43)	4.33 (4.23 to 4.44)	4.20 (4.09 to 4.30)	4.27 (4.15 to 4.40)
Will to compete, 1–5	4.23 (4.11 to 4.35)	4.36 (4.27 to 4.46)	4.19 (4.10 to 4.27)	4.19 (4.08 to 4.30)	4.38 (4.27 to 4.49)	4.52 (4.44 to 4.60)	4.34 (4.26 to 4.41)	4.34 (4.23 to 4.45)
**Achievement and Competitiveness Motivation**								
Mastery, 1–5	4.05 (3.95 to 4.15)	4.05 (3.97 to 4.13)	4.05 (3.97 to 4.13)	4.10 (4.00 to 4.20)	4.26 (4.17 to 4.35)	4.26 (4.20 to 4.33)	4.26 (4.19 to 4.32)	4.31 (4.22 to 4.41)
Work, 1–5	4.24 (4.12 to 4.35)	4.33 (4.25 to 4.41)	4.26 (4.18 to 4.34)	4.17 (4.06 to 4.28)	4.50 (4.39 to 4.59)	4.59 (4.52 to 4.66)	4.52 (4.46 to 4.59)	4.44 (4.32 to 4.54)
Competitiveness, 1–5	3.51 (3.35 to 3.66)	3.47 (3.36 to 3.58)	3.64 (3.54 to 3.75)	3.71 (3.57 to 3.85)	3.78 (3.63 to 3.92)	3.74 (3.65 to 3.83)	3.91 (3.82 to 4.00)	3.98 (3.84 to 4.13)
**Sources of Enjoyments in Youth Sports**								
Self-referenced competencies, 1–5	4.39 (4.28 to 4.49)	4.37 (4.29 to 4.45)	4.40 (4.32 to 4.49)	4.35 (4.25 to 4.45)	4.53 (4.44 to 4.62)	4.52 (4.45 to 4.58)	4.55 (4.48 to 4.61)	4.50 (4.41 to 4.58)
Others-referenced competencies, 1–5	3.44 (3.20 to 3.65)	3.61 (3.48 to 3.73)	3.64 (3.51 to 3.77)	3.75 (3.57 to 3.94)	3.66 (3.44 to 3.86)	3.83 (3.73 to 3.93)	3.86 (3.76 to 3.96)	3.97 (3.81 to 4.15)
Effort expenditure, 1–5	4.66 (4.54 to 4.77)	4.75 (4.68 to 4.82)	4.73 (4.66 to 4.80)	4.74 (4.65 to 4.82)	4.66 (4.54 to 4.75)	4.74 (4.69 to 4.80)	4.72 (4.66 to 4.77)	4.73 (4.66 to 4.81)
Affiliation with peers, 1–5	4.39 (4.24 to 4.52)	4.50 (4.41 to 4.60)	4.45 (4.36 to 4.54)	4.44 (4.32 to 4.55)	4.41 (4.27 to 4.53)	4.52 (4.45 to 4.60)	4.47 (4.39 to 4.54)	4.46 (4.35 to 4.55)
Positive parental involvement, 1–5	4.53 (4.37 to 4.70)	4.57 (4.46 to 4.68)	4.30 (4.18 to 4.42)	4.49 (4.34 to 4.65)	4.43 (4.28 to 4.59)	4.47 (4.38 to 4.57)	4.20 (4.10 to 4.30)	4.40 (4.26 to 4.53)

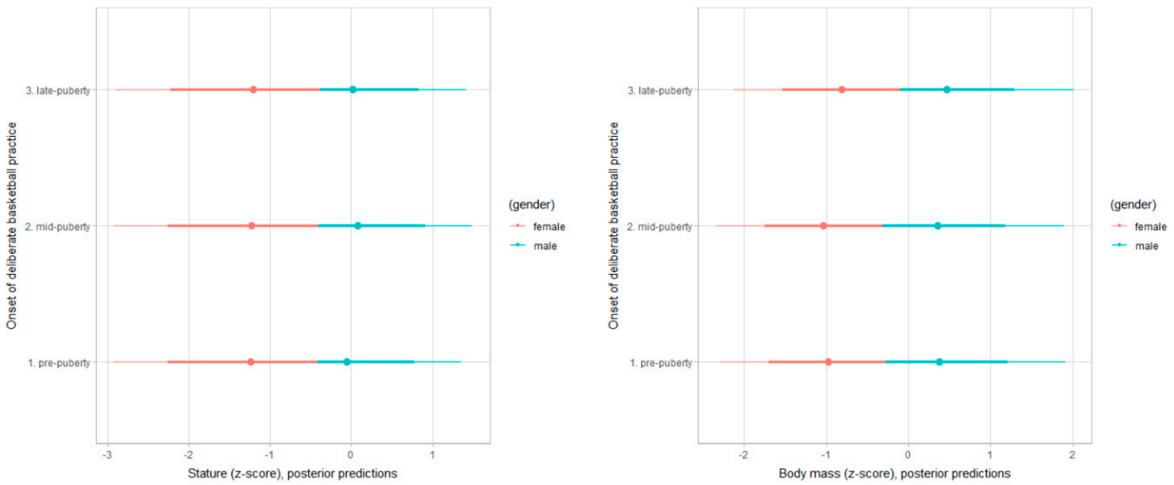

(a) **(b)**

Figure 1. Posterior predictions for stature **(a)** and body mass **(b)** by onset of deliberate basketball practice in young female and male basketball players (80% and 50% credible intervals).

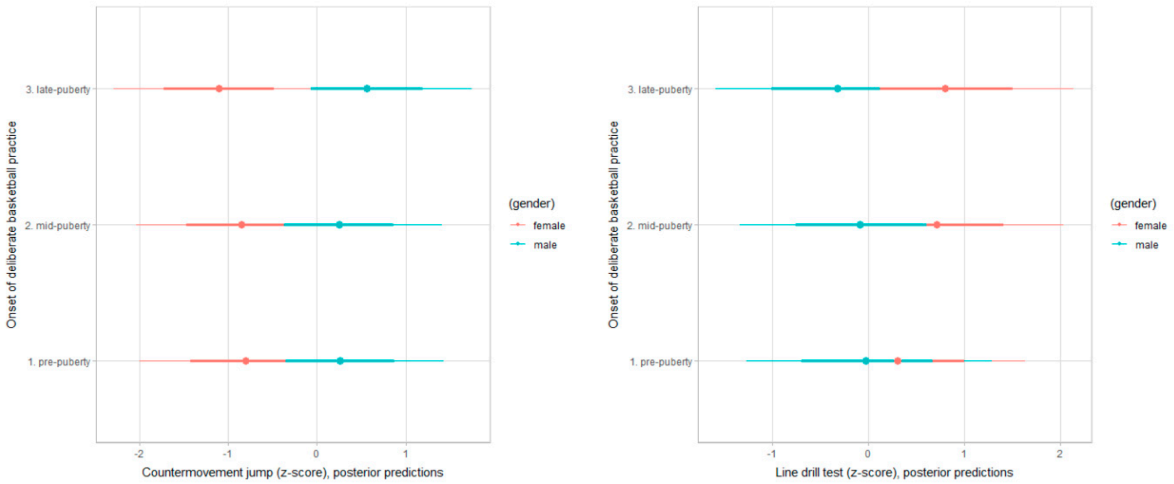

(a) **(b)**

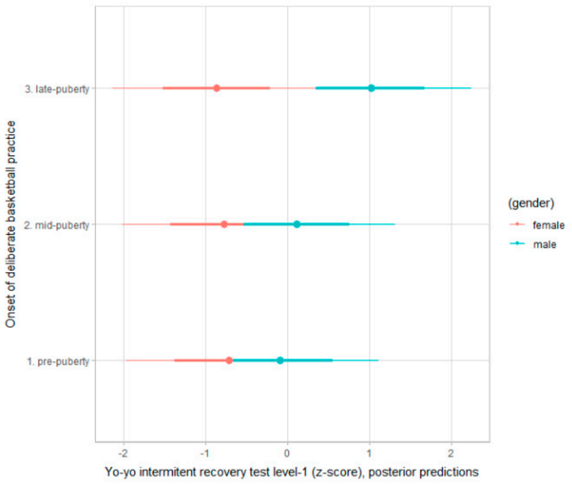

(c)

Figure 2. Posterior predictions for countermovement jump **(a)**, Line drill test **(b)** and yo-yo intermittent recovery test level-1 **(c)** by onset of deliberate basketball practice in young female and male basketball players (80% and 50% credible intervals).

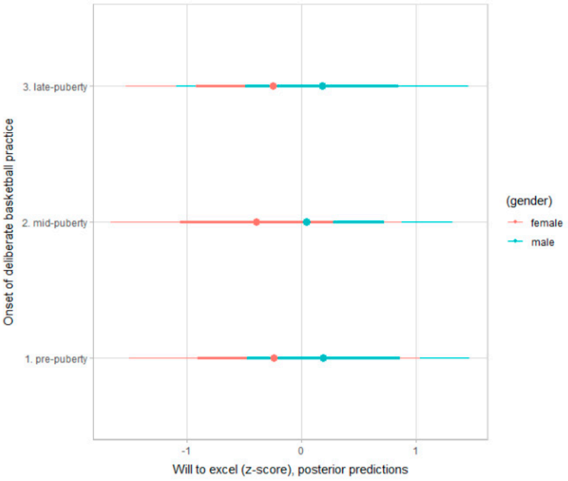

(a)

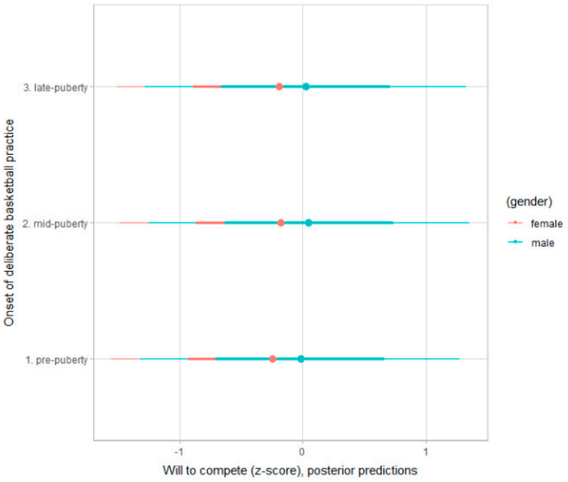

(b)

Figure 3. Posterior predictions for will to excel **(a)** and will to compete **(b)** by onset of deliberate basketball practice in young female and male basketball players (80% and 50% credible intervals).

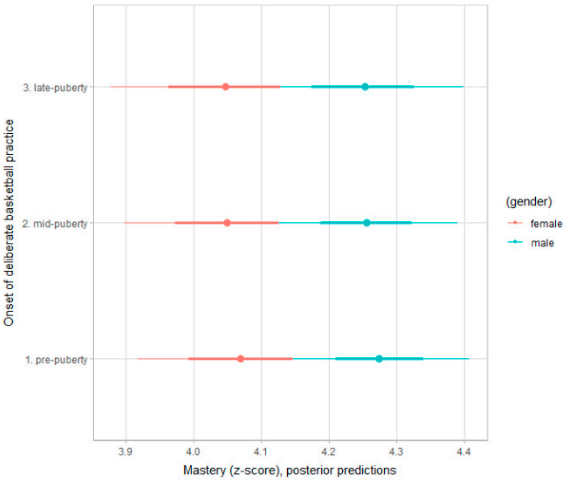

(a)

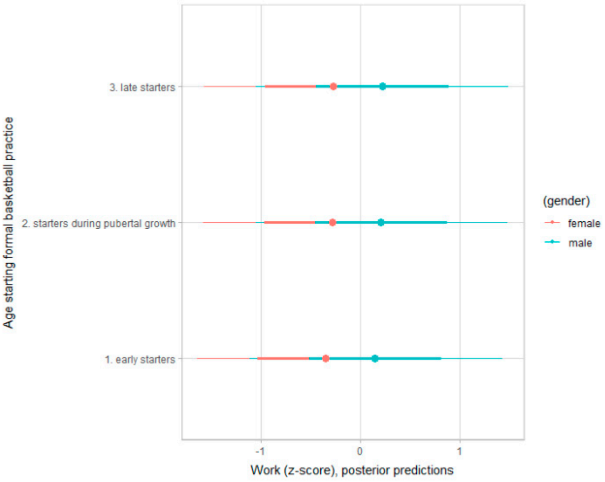

(b)

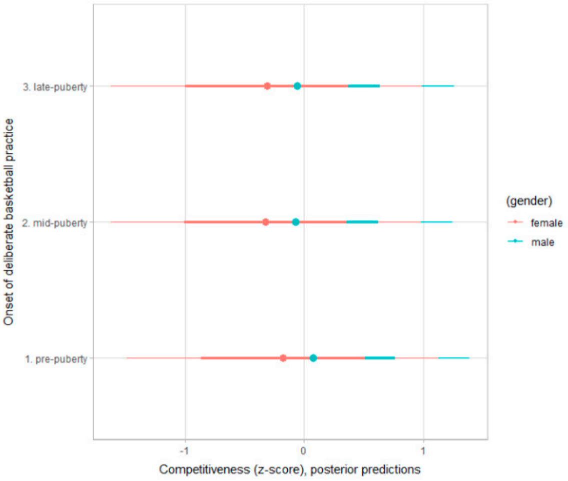

(c)

Figure 4. Posterior predictions for mastery **(a)**, work **(b)** and competitiveness **(c)** by onset of deliberate basketball practice in young female and male basketball players (80% and 50% credible intervals).

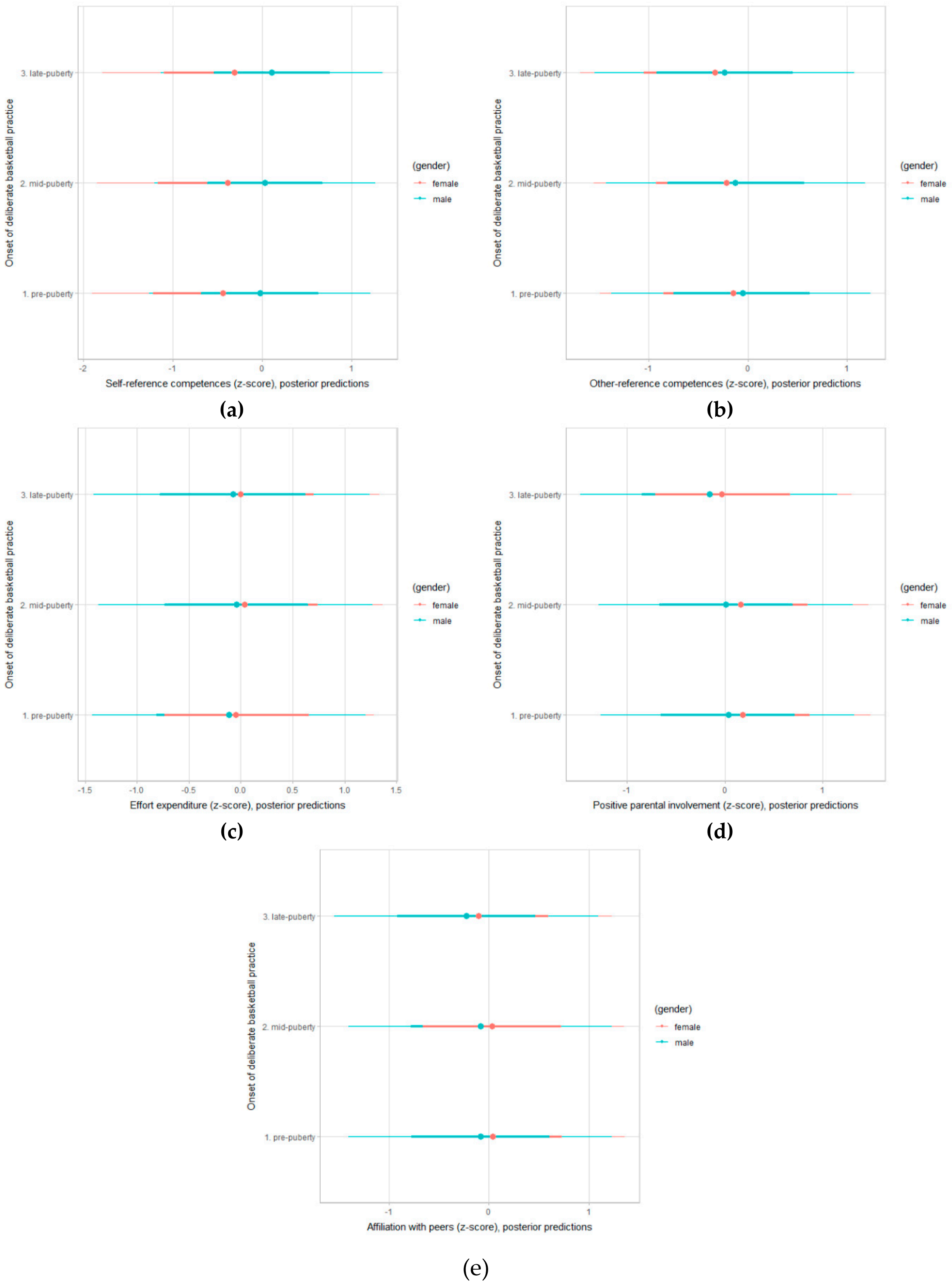

Figure 5. Posterior predictions for self-referenced competences **(a)**, others-referenced competences **(b)**, effort expenditure **(c)**, positive parental involvement **(d)** and affiliation with peers **(e)** by onset of deliberate basketball practice in young female and male basketball players (80% and 50% credible intervals).

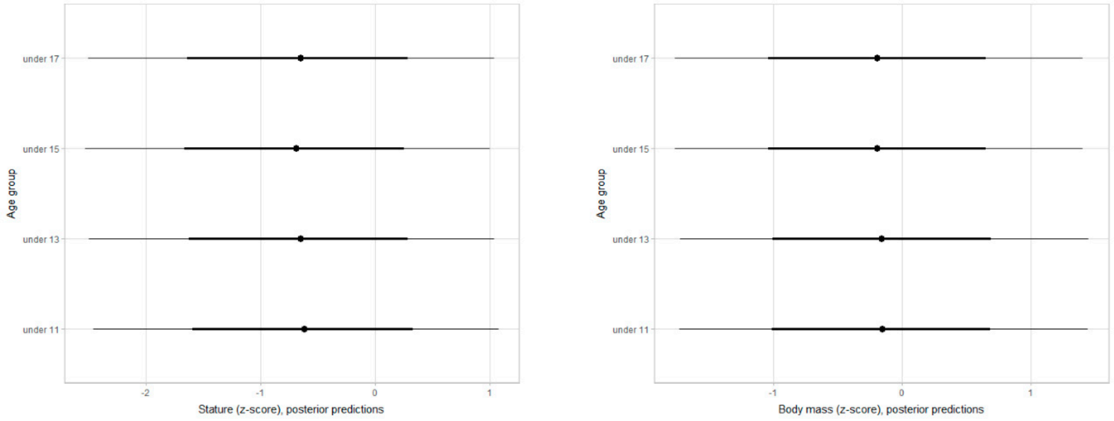

(a) **(b)**

Figure 6. Posterior predictions for stature **(a)** and body mass **(b)** by age group in young female and male basketball players, adjusting for maturity status, age starting basketball practice and state basketball federation (80% and 50% credible intervals).

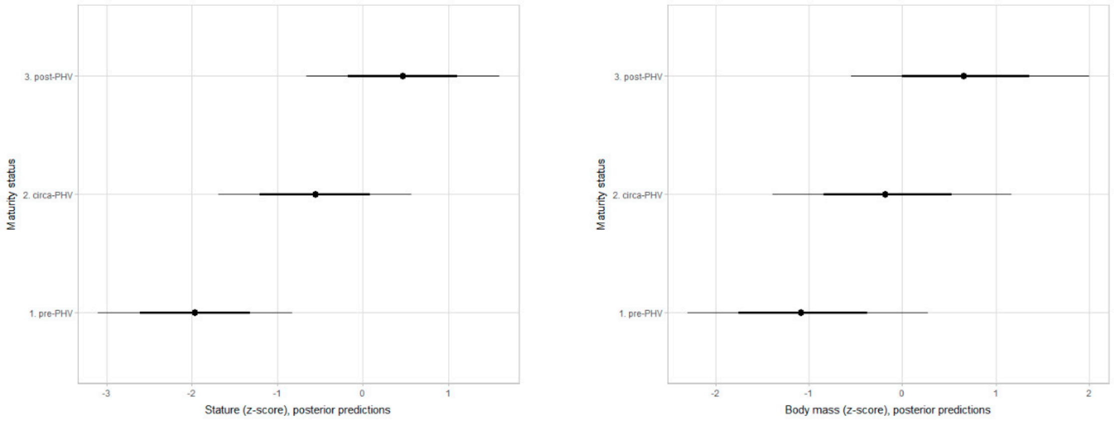

(a) **(b)**

Figure 7. Posterior predictions for stature **(a)** and body mass **(b)** by maturity status in young female and male basketball players, adjusting for age group, age starting basketball practice and state basketball federation (80% and 50% credible intervals).

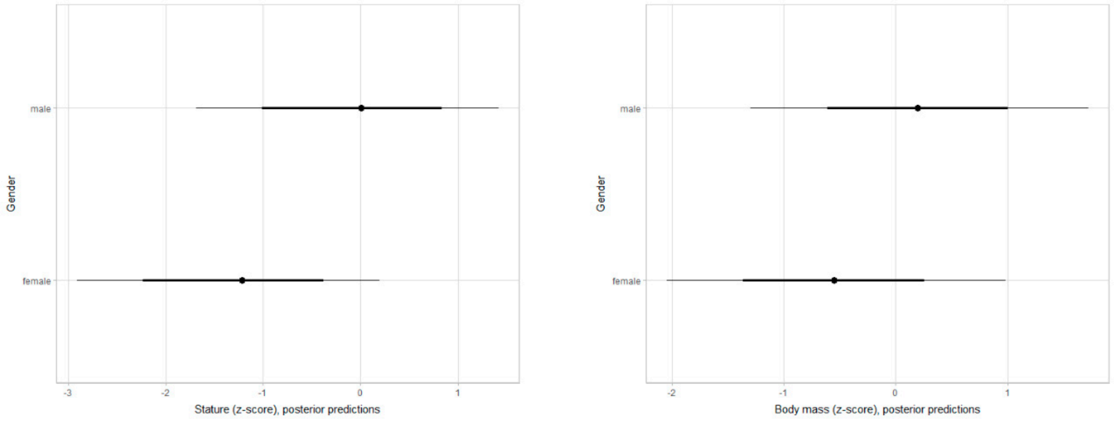

(a) **(b)**

Figure 8. Posterior predictions for stature **(a)** and body mass **(b)** in young female and male basketball players, adjusting for age group, maturity status, age starting basketball practice and state basketball federation (80% and 50% credible intervals).

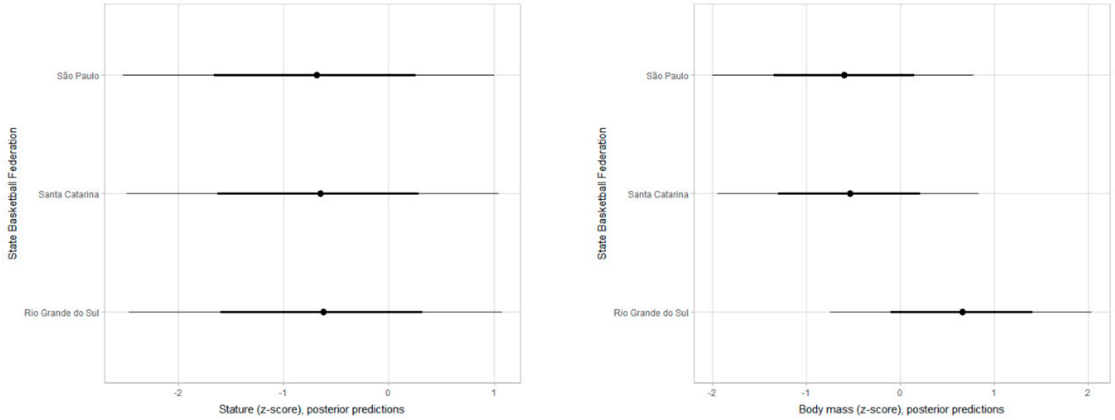

(a) **(b)**

Figure 9. Posterior predictions for stature **(a)** and body mass **(b)** by state basketball federation in young female and male basketball players, adjusting for age group, maturity status and age starting basketball practice (80% and 50% credible intervals).

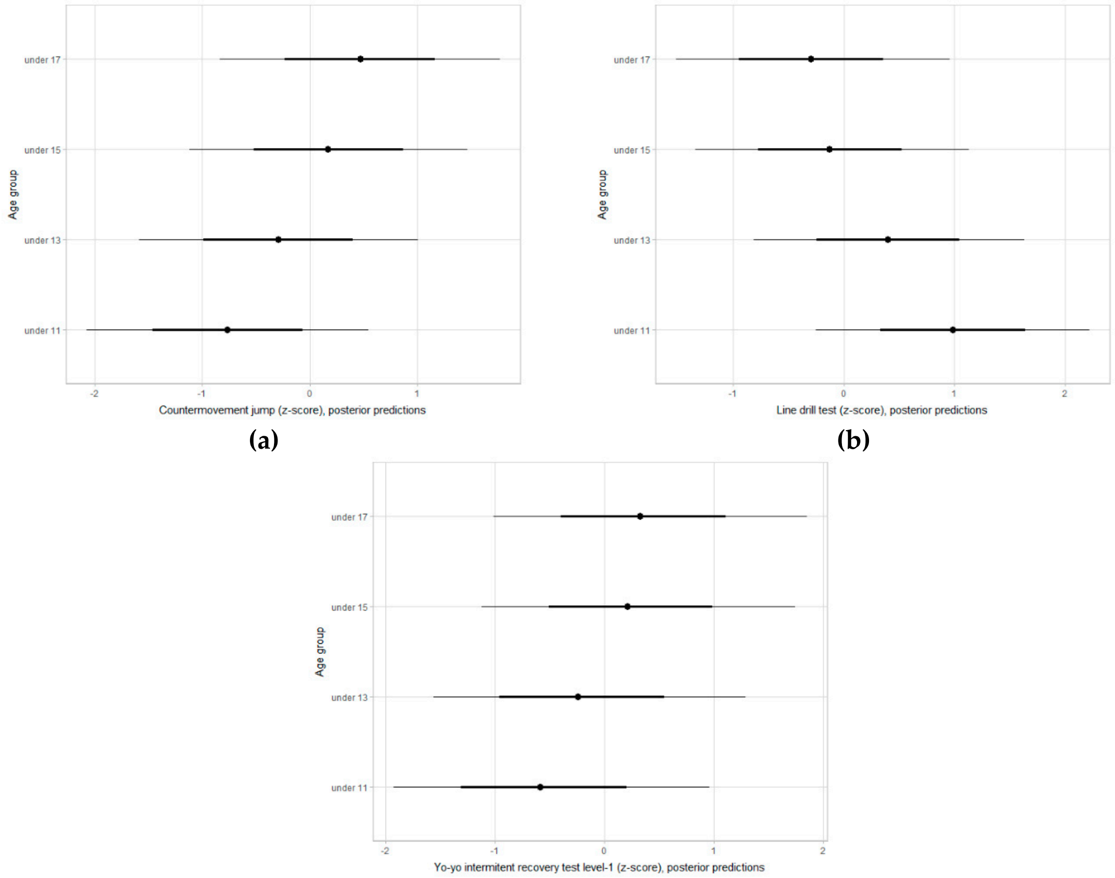

(a) **(b)**

(c)

Figure 10. Posterior predictions for countermovement jump **(a)**, Line drill test **(b)** and yo-yo intermittent recovery test level-1 **(c)** by age group in young female and male basketball players, adjusting for maturity status, age starting basketball practice and state basketball federation (80% and 50% credible intervals).

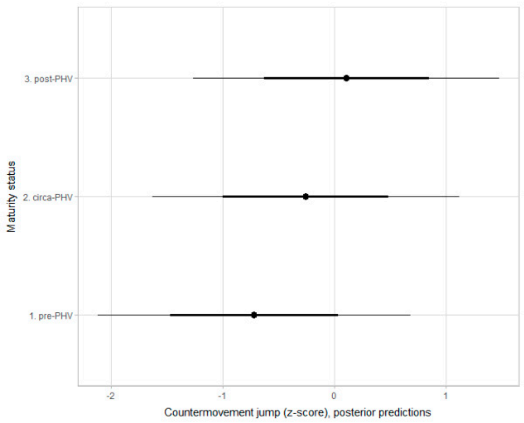

(a)

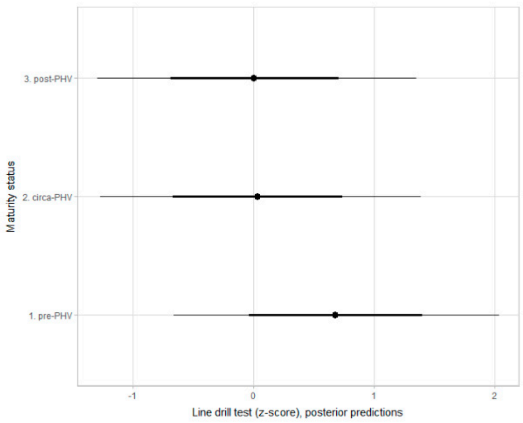

(b)

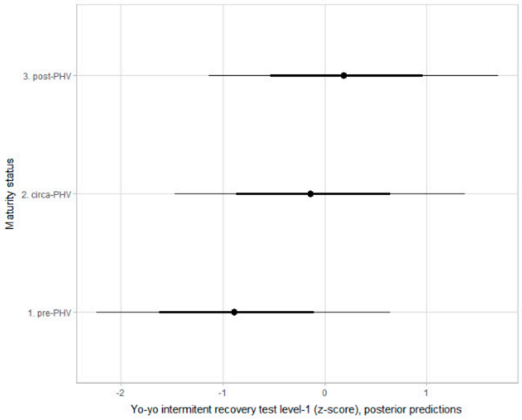

(c)

Figure 11. Posterior predictions for countermovement jump **(a)**, Line drill test **(b)** and yo-yo intermittent recovery test level-1 **(c)** by maturity status in young female and male basketball players, adjusting for age group, age starting basketball practice and state basketball federation (80% and 50% credible intervals).

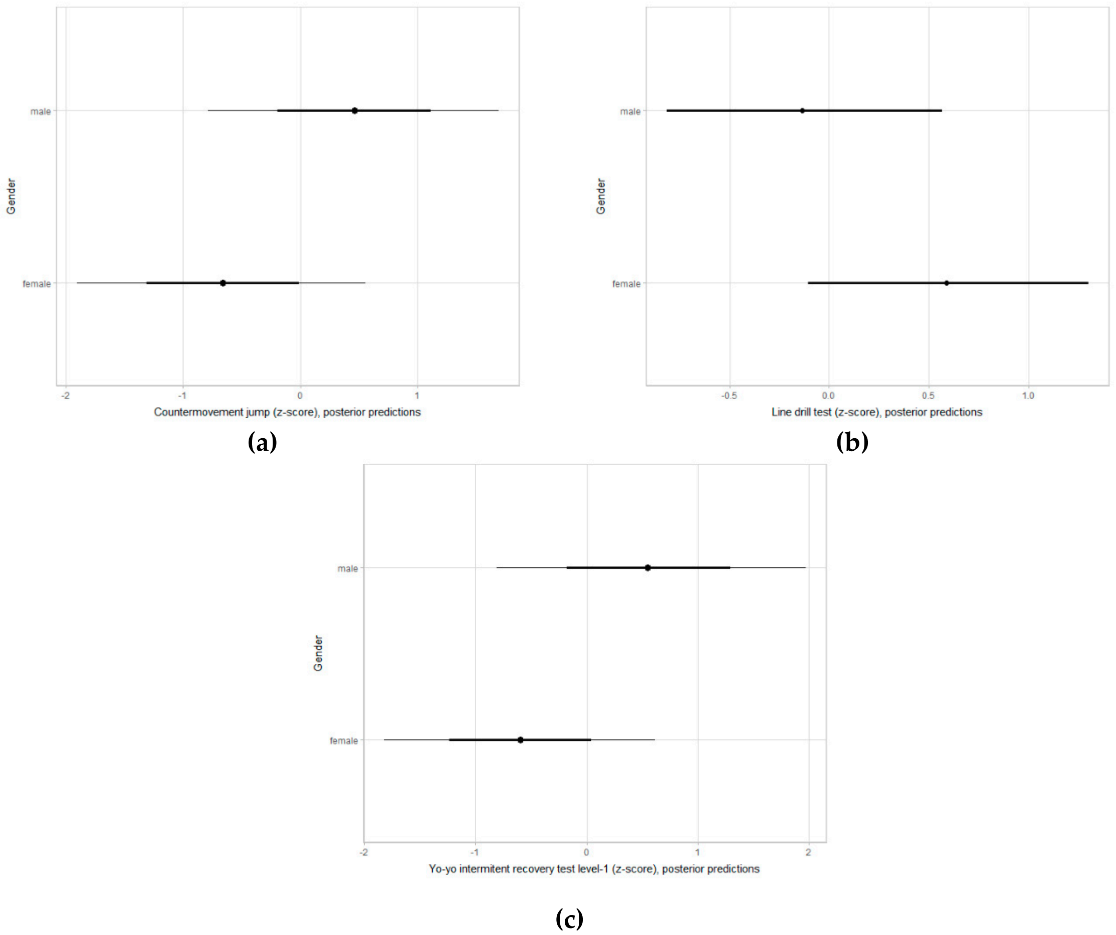

Figure 12. Posterior predictions for countermovement jump **(a)**, Line drill test **(b)** and yo-yo intermittent recovery test level-1 **(c)** in young female and male basketball players, adjusting for age group, maturity status, age starting basketball practice and state basketball federation (80% and 50% credible intervals).

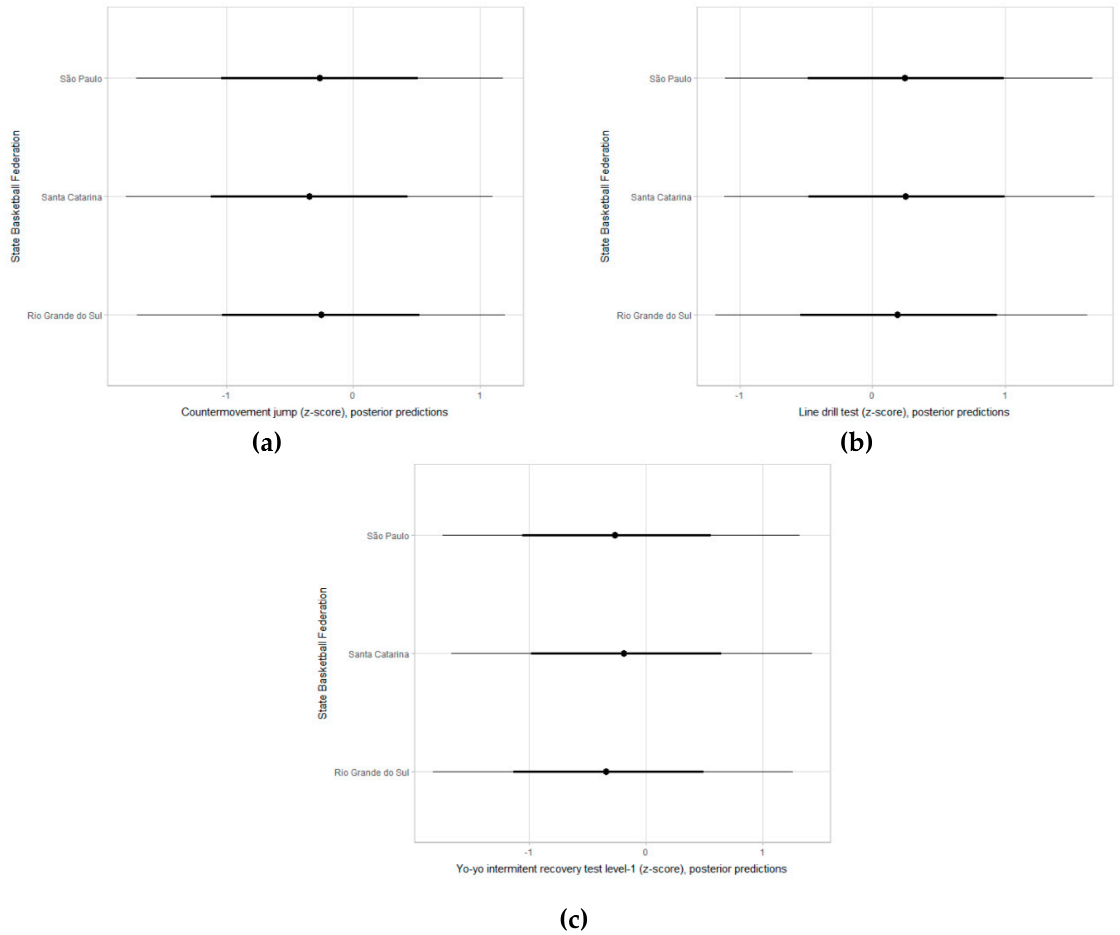

Figure 13. Posterior predictions for countermovement jump **(a)**, Line drill test **(b)** and yo-yo intermittent recovery test level-1 **(c)** by state basketball federation in young female and male basketball players, adjusting for age group, maturity status and age starting basketball practice (80% and 50% credible intervals).

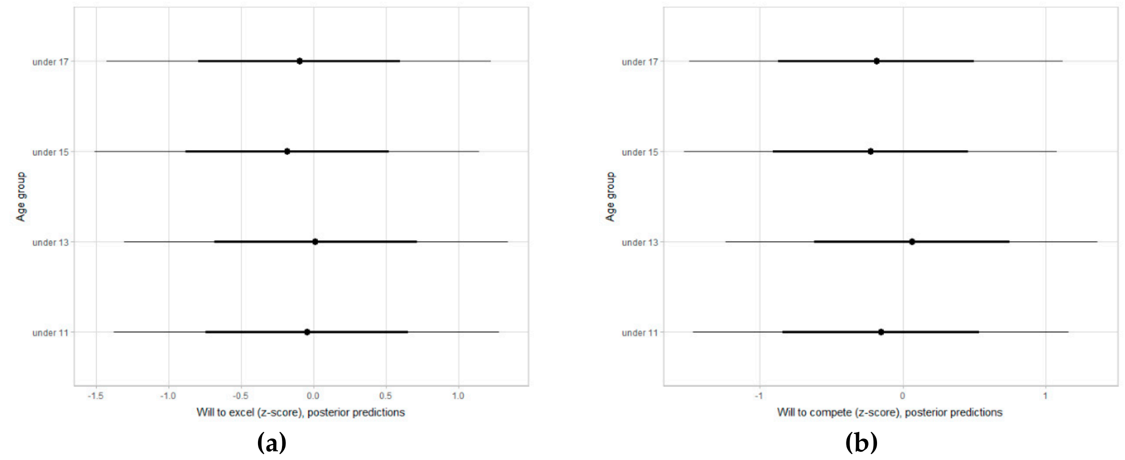

Figure 14. Posterior predictions for will to excel **(a)** and will to compete **(b)** by age group in young female and male basketball players, adjusting for maturity status, age starting basketball practice and state basketball federation (80% and 50% credible intervals).

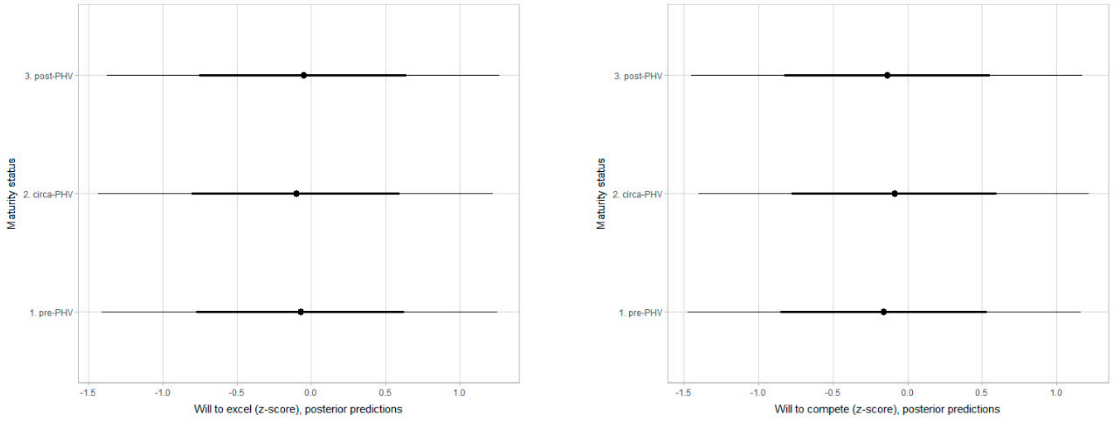

(a) **(b)**

Figure 15. Posterior predictions for will to excel **(a)** and will to compete **(b)** by maturity status in young female and male basketball players, adjusting for age group, age starting basketball practice and state basketball federation (80% and 50% credible intervals).

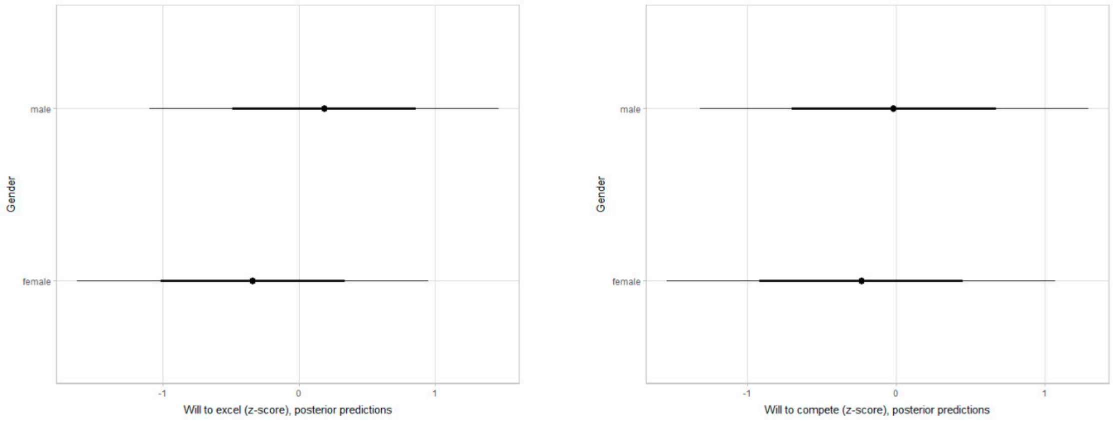

(a) **(b)**

Figure 16. Posterior predictions for deliberate practice motivation dimensions in young female and male basketball players, adjusting for age group, maturity status, age starting basketball practice and state basketball federation (80% and 50% credible intervals).

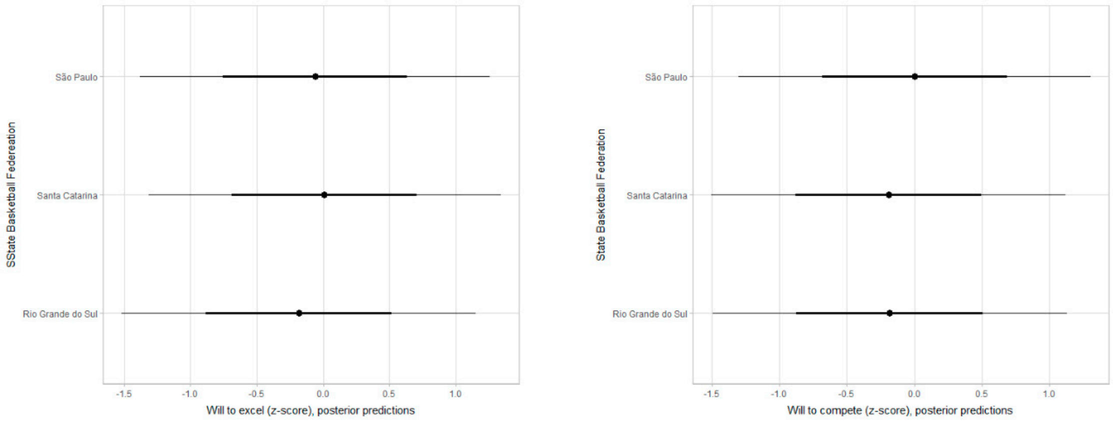

(a) **(b)**

Figure 17. Posterior predictions for will to excel **(a)** and will to compete **(b)** by state basketball federation in young female and male basketball players, adjusting for age group, maturity status and age starting basketball practice (80% and 50% credible intervals).

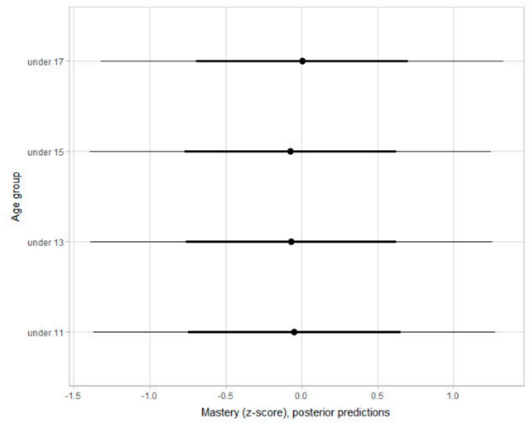

(a)

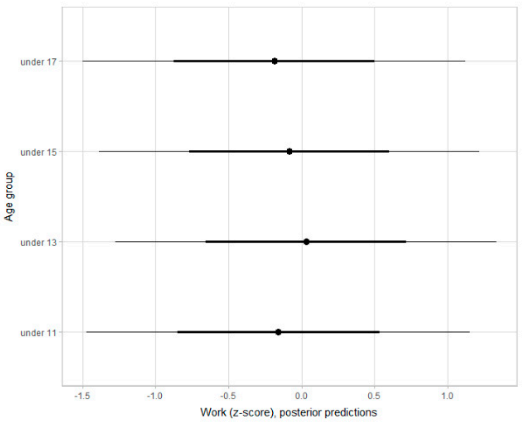

(b)

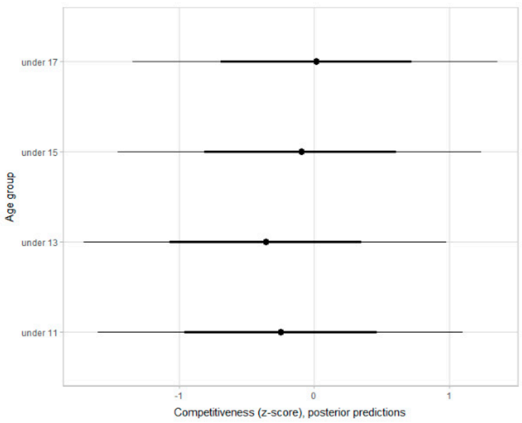

(c)

Figure 18. Posterior predictions for mastery **(a),** work **(b)** and competitiveness **(c)** by age group in young female and male basketball players, adjusting for maturity status, age starting basketball practice and state basketball federation (80% and 50% credible intervals).

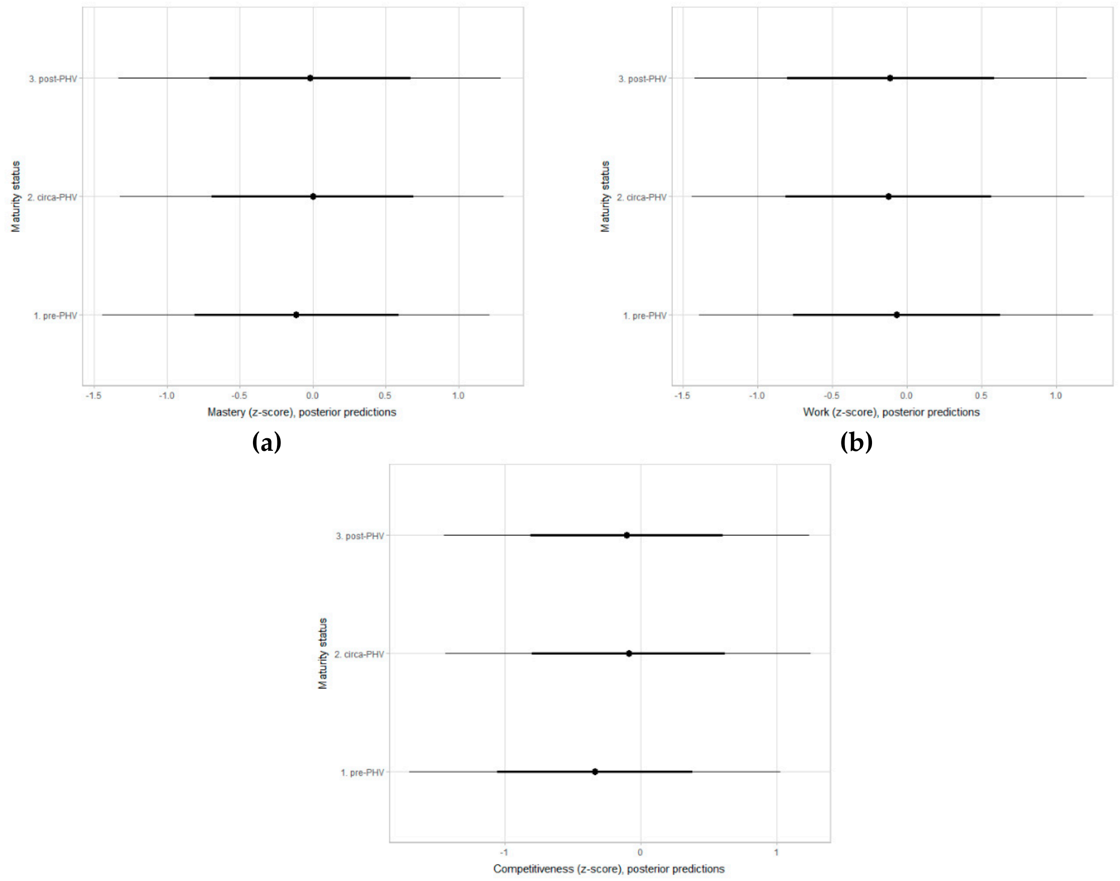

(a) (b) (c)

Figure 19. Posterior predictions for mastery **(a),** work **(b)** and competitiveness **(c)** by maturity status in young female and male basketball players, adjusting for age group, age starting basketball practice and state basketball federation (80% and 50% credible intervals).

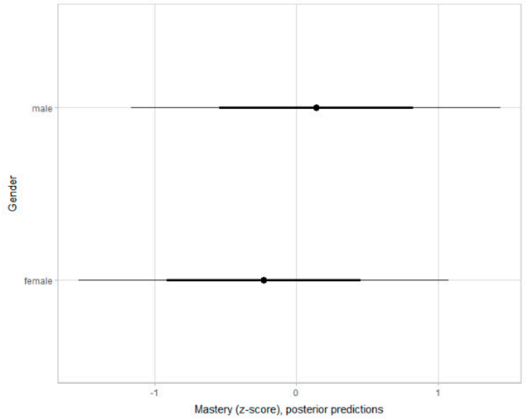

(a)

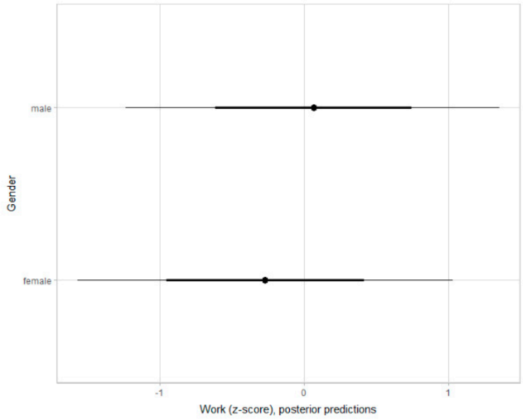

(b)

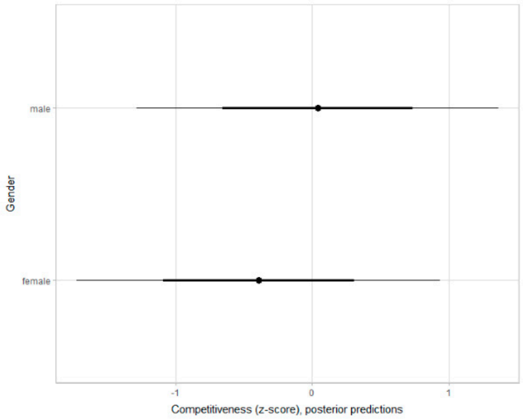

(c)

Figure 20. Posterior predictions for mastery **(a),** work **(b)** and competitiveness **(c)** in young female and male basketball players, adjusting for age group, maturity status, age starting basketball practice and state basketball federation (80% and 50% credible intervals).

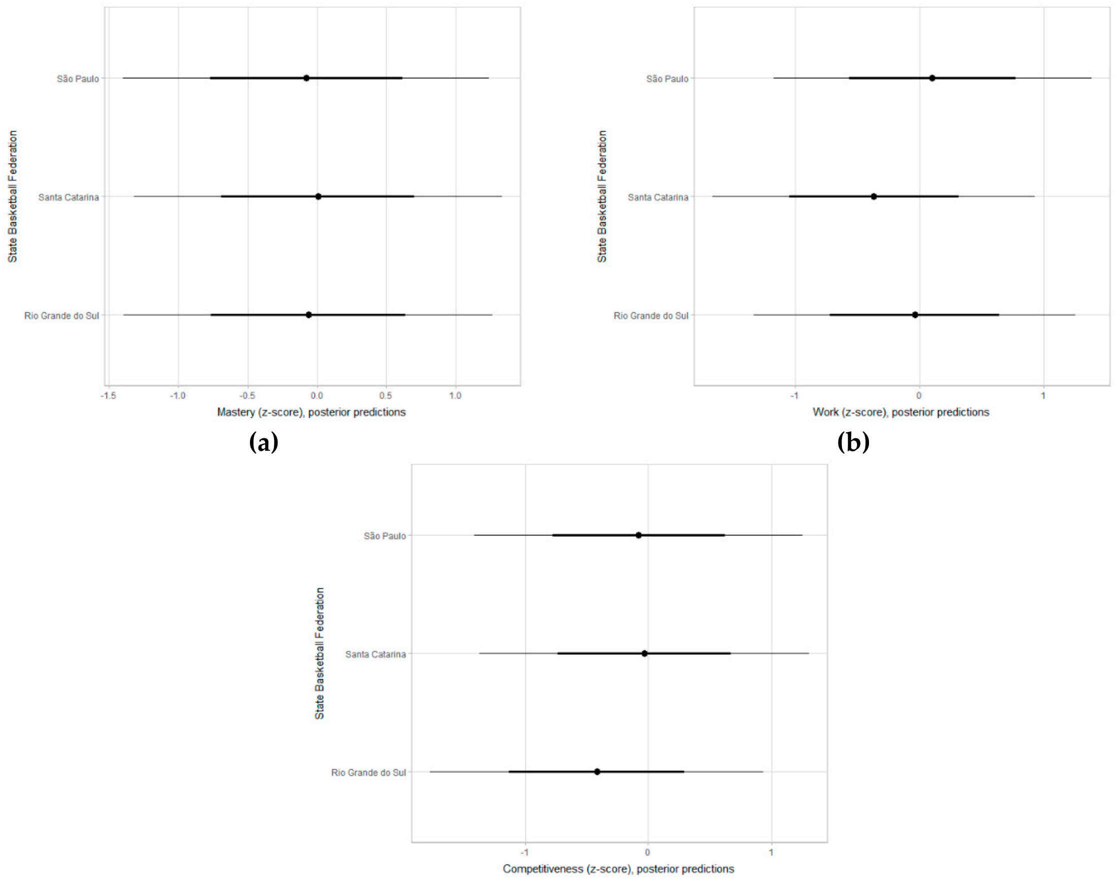

(c)

Figure 21. Posterior predictions for mastery **(a),** work **(b)** and competitiveness **(c)** by state basketball federation in young female and male basketball players, adjusting for age group, maturity status and age starting basketball practice (80% and 50% credible intervals).

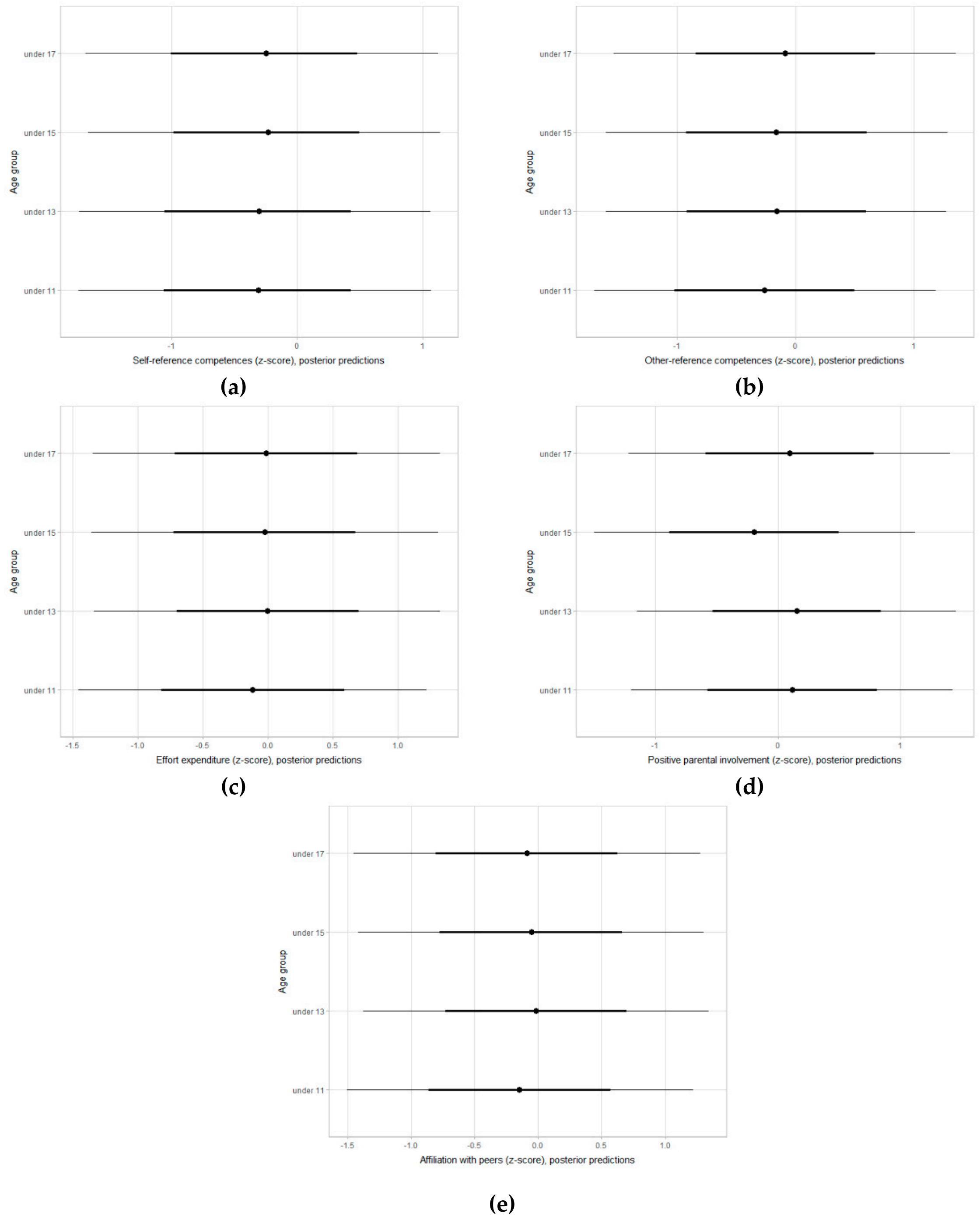

Figure 22. Posterior predictions for self-referenced competences **(a)**, others-referenced competences **(b)**, effort expenditure **(c)**, positive parental involvement **(d)** and affiliation with peers **(e)** by age group in young female and male basketball players, adjusting for maturity status, age starting basketball practice and state basketball federation (80% and 50% credible intervals).

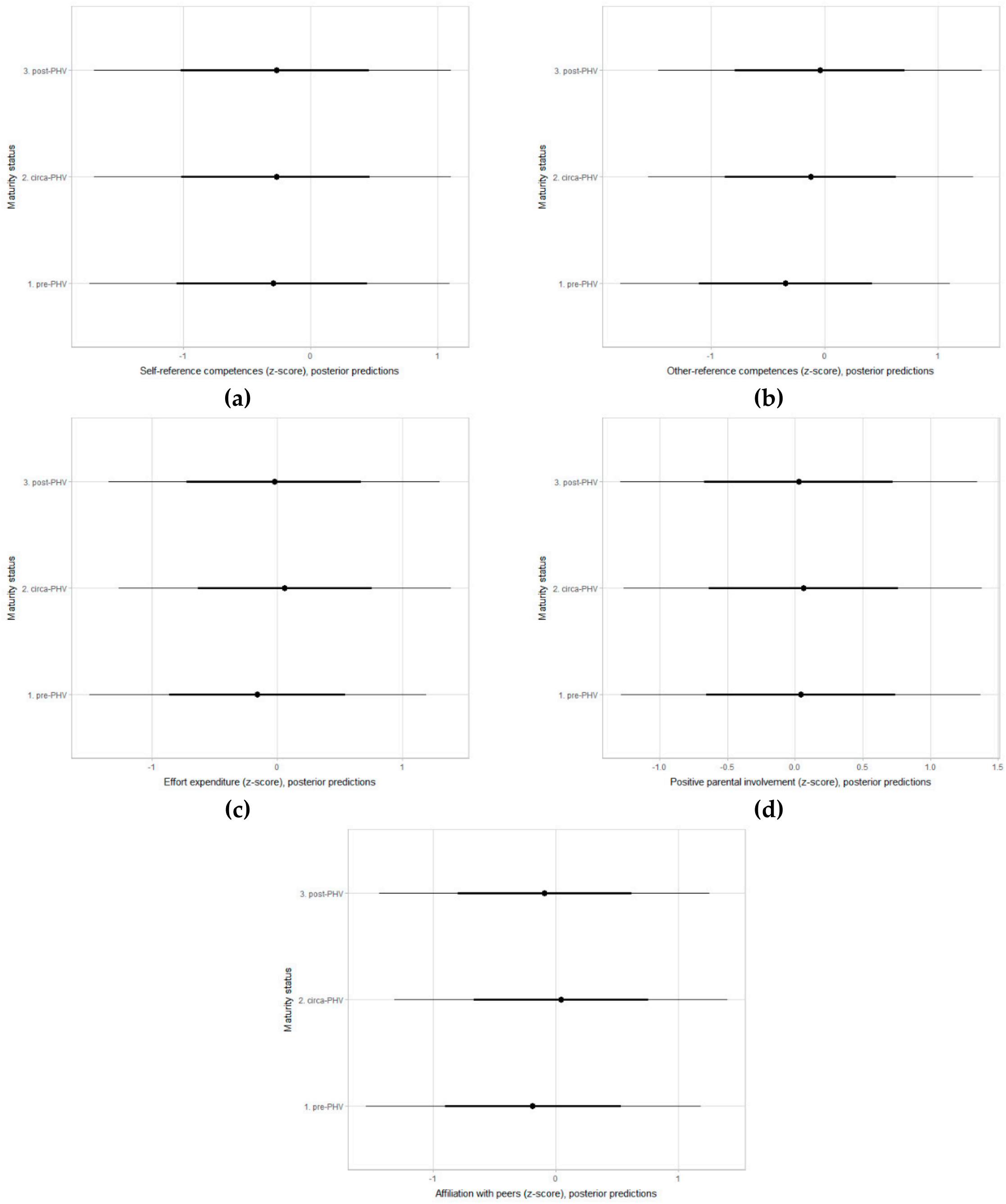

(e)

Figure 23. Posterior predictions for self-referenced competences **(a)**, others-referenced competences **(b)**, effort expenditure **(c)**, positive parental involvement **(d)** and affiliation with peers **(e)** by maturity status in young female and male basketball players, adjusting for age group, age starting basketball practice and state basketball federation (80% and 50% credible intervals).

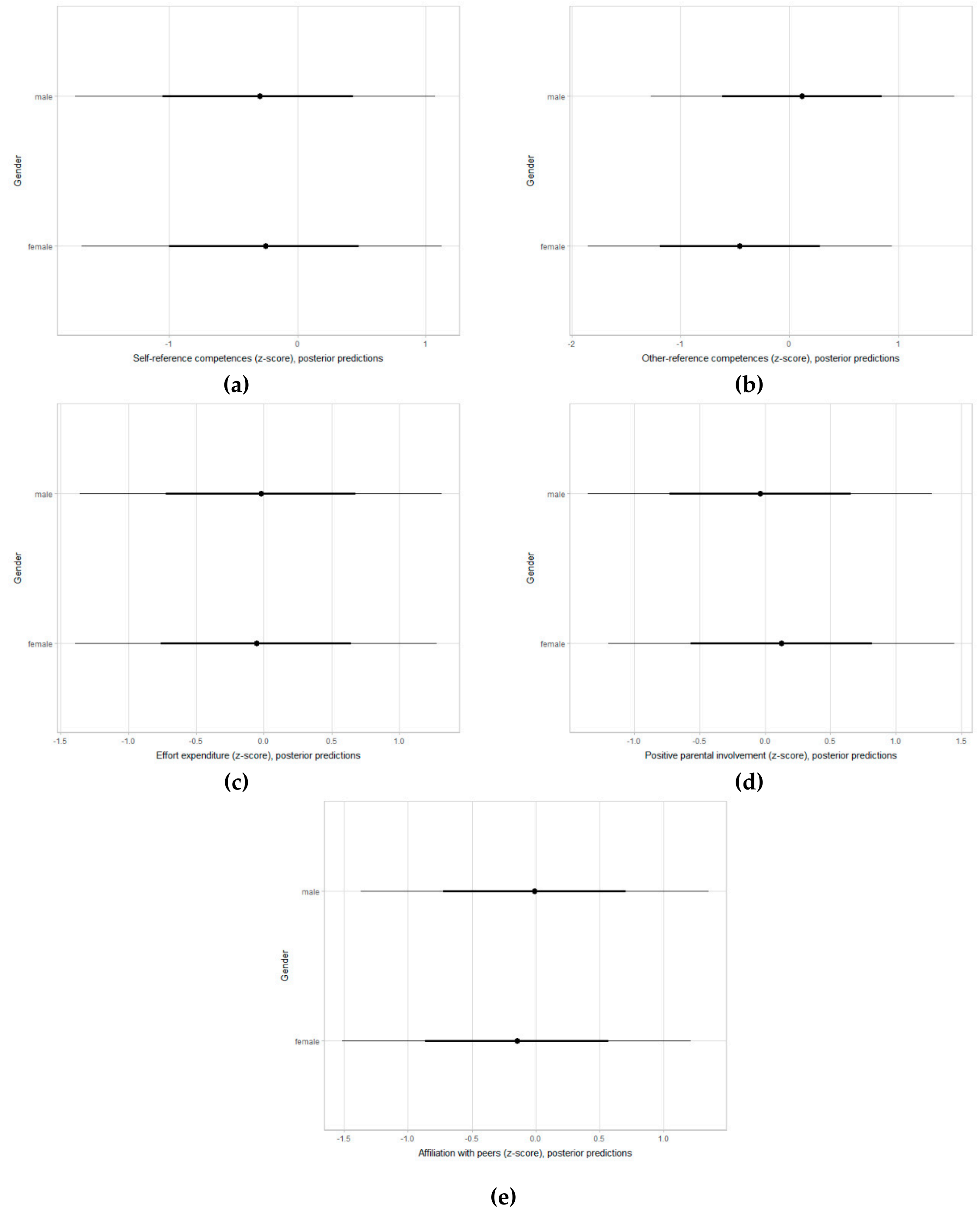

Figure 24. Posterior predictions for self-referenced competences **(a)**, others-referenced competences **(b)**, effort expenditure **(c)**, positive parental involvement **(d)** and affiliation with peers **(e)** in young female and male basketball players, adjusting for age group, maturity status, age starting basketball practice and state basketball federation (80% and 50% credible intervals).

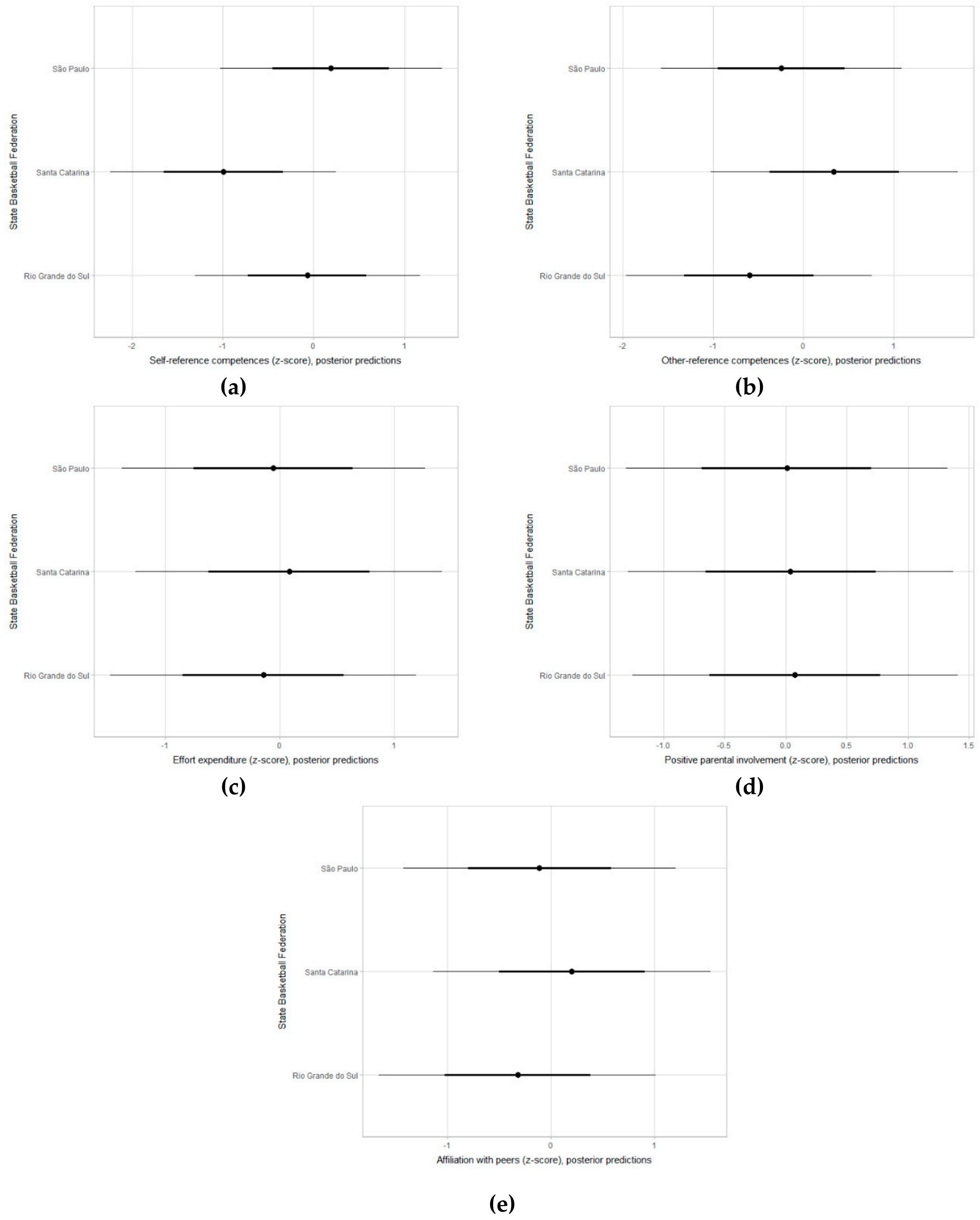

Figure 25. Posterior predictions for self-referenced competences **(a)**, others-referenced competences **(b)**, effort expenditure **(c)**, positive parental involvement **(d)** and affiliation with peers **(e)** by state basketball federation in young female and male basketball players, adjusting for age group maturity status and age starting basketball practice (80% and 50% credible intervals).

Codes for all the models in the manuscript:

```
library(magrittr)
library(dplyr)
library(purrr)
library(forcats)
library(tidyr)
library(modelr)
library(tidybayes)
library(ggplot2)
library(ggstance)
library(ggridges)
library(cowplot)
library(rstan)
library(brms)
library(ggrepel)
library(RColorBrewer)
library(gganimate)

theme_set(theme_tidybayes() + panel_border() + background_grid())

rstan_options(auto_write = TRUE)
options(mc.cores = parallel::detectCores())

#meta analysis

aphv1<brm(mean_age_PHV|se(se)~1+(1|id)+(1|gender),
data = meta_APHV,
family = "gaussian",
prior = c(prior(normal(0,2),class = sd)),
control = list(adapt_delta = 0.99,max_treedepth = 15))
```

```
iphv1 < brm(mean_age_initiation_PHV|se(se)~1+(1|id)+(1|gender),

data = meta_IPHV,

family = "gaussian",
prior = c(prior(normal(0,2),class=sd)),
control = list(adapt_delta = 0.99,max_treedepth = 15))

#performance

m1<brm(yoyo_s~(1|age_group)+(1|specialization)+(1|phv_cat)+(1|gender)+(1|state)+(1|specializatio
    n:gender),
    data = bball, family = gaussian,
    prior = c(prior(normal(0,2),class = Intercept),prior(normal(0,2),class = sd)),
    chains = 4, iter = 2000, warmup = 1000, cores = 4,
    control = list(adapt_delta=0.99,max_treedepth=15))

plot(m1)
pp_ckeck(m1)
summary(m1)
coef(m1)

yoyo.special.sex.s<-bball %>%
    group_by(gender) %>%
    data_grid(age_group,specialization,gender, mat_sample,state) %>%
    add_predicted_draws(m1) %>%
    ggplot(aes(x = .prediction, y = specialization,color = (gender))) +
    stat_pointintervalh(.width = c(.8,.5))+ labs(y= "Specialization")+ labs(x= "Yo-yo intermitent recovery
    test level-1 (z-score), posterior predictions")

yoyo.sex.s<-bball %>%
    data_grid(age_group,specialization,gender, mat_sample,state) %>%
    add_predicted_draws(m1) %>%
    ggplot(aes(x = .prediction, y = gender)) +
    stat_pointintervalh(.width = c(.8,.5))+ labs(y= "Gender")+ labs(x= "Yo-yo intermitent recovery test
    level-1 (z-score), posterior predictions")

yoyo.age.s<-bball %>%
data_grid(age_group,specialization,gender, mat_sample,state) %>%
add_predicted_draws(m1) %>%
ggplot(aes(x = .prediction, y = age_group)) +
stat_pointintervalh(.width = c(.8,.5))+ labs(y = "Age group")+ labs(x= "Yo-yo intermitent recovery test
    level-1 (z-score), posterior predictions")

yoyo.mat.s<-bball %>%
data_grid(age_group,specialization,gender, phv_cat,state) %>%
add_predicted_draws(m1) %>%
ggplot(aes(x = .prediction, y = phv_cat)) +
stat_pointintervalh(.width = c(.8,.5))+ labs(y = "Maturity status")+ labs(x = "Yo-yo intermitent recovery
    test level-1 (z-score), posterior predictions")

yoyo.state.s<-bball %>%
```

```
data_grid(age_group,specialization,gender, phv_cat,state) %>%
add_predicted_draws(m1) %>%
ggplot(aes(x = .prediction, y = state)) +
stat_pointintervalh(.width = c(.8,.5))+ labs(y = "State Basketball Federation")+ labs(x= "Yo-yo intermitent
    recovery test level-1 (z-score), posterior predictions")

yoyo.special.state.s<-bball %>%
data_grid(age_group,specialization,gender, phv_cat,state) %>%
add_predicted_draws(m1) %>%
ggplot(aes(x = .prediction, y = state, color = (specialization))) +
stat_pointintervalh(.width = c(.8,.5))+ labs(y = "State Basketball Federation")+ labs(x = "Yo-yo
    intermitent recovery test level-1 (z-score), posterior predictions")

m2<-
    brm(ld_s~(1|age_group)+(1|specialization)+(1|phv_cat)+(1|gender)+(1|state)+(1|specialization:ge
    nder),
    data = bball,
    family = gaussian, prior = c(prior(normal(0,1),class = Intercept),prior(normal(0,1),class = sd)),
    chains = 4, iter = 2000, warmup = 1000, cores = 4,
    control = list(adapt_delta = 0.99,max_treedepth = 15))

plot(m2)
pp_ckeck(m2)
summary(m2)
coef(m2)

ld.special.sex.s<-bball %>%
group_by(gender) %>%
data_grid(age_group,specialization,gender, mat_sample,state) %>%
add_predicted_draws(m2) %>%
ggplot(aes(x = .prediction, y = specialization,color = (gender))) +
stat_pointintervalh(.width = c(.8,.5))+ labs(y = "Specialization")+ labs(x = "Line drill test (z-score),
    posterior predictions")

ld.sex.s<-bball %>%
data_grid(age_group,specialization,gender, phv_cat,state) %>%
add_predicted_draws(m2) %>%
ggplot(aes(x = .prediction, y = gender)) +
stat_pointintervalh(.width = c(.8,.5))+ labs(y = "Gender")+ labs(x = "Line drill test (z-score), posterior
    predictions")

ld.age.s<-bball %>%
 data_grid(age_group,specialization,gender, phv_cat,state) %>%
add_predicted_draws(m2) %>%
ggplot(aes(x = .prediction, y = age_group)) +
stat_pointintervalh(.width = c(.8,.5))+ labs(y = "Age group")+ labs(x = "Line drill test (z-score), posterior
    predictions")

ld.mat.s<-bball %>%
data_grid(age_group,specialization,gender, phv_cat,state) %>%
add_predicted_draws(m2) %>%
```

```
ggplot(aes(x = .prediction, y = phv_cat)) +
stat_pointintervalh(.width = c(.8,.5))+ labs(y = "Maturity status")+ labs(x = "Line drill test (z-score),
    posterior predictions")

ld.state.s<-bball %>%
data_grid(age_group,specialization,gender, phv_cat,state) %>%
add_predicted_draws(m2) %>%
ggplot(aes(x = .prediction, y = state)) +
stat_pointintervalh(.width = c(.8,.5))+ labs(y = "State Basketball Federation")+ labs(x = "Line drill test (z-
    score), posterior predictions")

ld.special.state.s<-bball %>%
data_grid(age_group,specialization,gender, phv_cat,state) %>%
add_predicted_draws(m2) %>%
ggplot(aes(x = .prediction, y = state, color = (specialization))) +
stat_pointintervalh(.width = c(.8,.5))+ labs(y = "State Basketball Federation")+ labs(x = "Line drill test (z-
    score), posterior predictions")

m3<brm(jump_s~(1|age_group)+(1|specialization)+(1|phv_cat)+(1|gender)+(1|state)+(1|specializatio
    n:gender),
    data = bball,
    family = Gaussian, prior = c(prior(normal(0,1),class = Intercept),prior(normal(0,1),class = sd)),
    chains = 4, iter = 2000, warmup = 1000, cores = 4,
    control = list(adapt_delta = 0.99,max_treedepth = 15))

plot(m3)
pp_ckeck(m3)
summary(m3)
coef(m3)

jump.special.sex.s<-bball %>%
group_by(gender) %>%
data_grid(age_group,specialization,gender, mat_sample,state) %>%
add_predicted_draws(m3) %>%
ggplot(aes(x = .prediction, y = specialization,color = (gender))) +
stat_pointintervalh(.width = c(.8,.5))+ labs(y = "Specialization")+ labs(x = "Countermovement jump (z-
    score), posterior predictions")

jump.sex.s<-bball %>%
data_grid(age_group,specialization,gender, mat_sample,state) %>%
add_predicted_draws(m3) %>%
ggplot(aes(x = .prediction, y = gender)) +
stat_pointintervalh(.width = c(.8,.5))+ labs(y = "Gender")+ labs(x = "Countermovement jump (z-score),
    posterior predictions")

jump.age.s<-bball %>%
data_grid(age_group,specialization,gender, mat_sample,state) %>%
add_predicted_draws(m3) %>%
ggplot(aes(x = .prediction, y = age_group)) +
stat_pointintervalh(.width = c(.8,.5))+ labs(y = "Age group")+ labs(x = "Countermovement jump (z-
    score), posterior predictions")
```

```
jump.mat.s<-bball %>%
data_grid(age_group,specialization,gender, phv_cat,state) %>%
add_predicted_draws(m3) %>%
ggplot(aes(x = .prediction, y = phv_cat)) +
stat_pointintervalh(.width = c(.8,.5))+ labs(y = "Maturity status")+ labs(x = "Countermovement jump (z-score), posterior predictions")

jump.state.s<-bball %>%
data_grid(age_group,specialization,gender, phv_cat,state) %>%
add_predicted_draws(m3) %>%
ggplot(aes(x = .prediction, y = state)) +
stat_pointintervalh(.width = c(.8,.5))+ labs(y = "State Basketball Federation")+ labs(x = "Countermovement jump (z-score), posterior predictions")

jump.special.state.s<-bball %>%
data_grid(age_group,specialization,gender, phv_cat,state) %>%
add_predicted_draws(m3) %>%
ggplot(aes(x = .prediction, y = state, color=(specialization))) +
stat_pointintervalh(.width = c(.8,.5))+ labs(y = "State Basketball Federation")+ labs(x = "Countermovement jump (z-score), posterior predictions")

###################
#body size

m4<-
brm(h_s~(1|age_group)+(1|specialization)+(1|phv_cat)+(1|gender)+(1|state)+(1|specialization:gender),
    data = bball,
    family = gaussian,
    prior = c(prior(normal(0,1),class = Intercept),prior(normal(0,1),class = sd)),
    chains = 4, iter = 2000, warmup = 1000, cores = 4,
    control = list(adapt_delta = 0.99,max_treedepth = 15))

plot(m4)
pp_ckeck(m4)
summary(m4)
coef(m4)

h.special.sex.s<-bball %>%
group_by(gender) %>%
data_grid(age_group,specialization,gender, phv_cat,state) %>%
add_predicted_draws(m4) %>%
ggplot(aes(x = .prediction, y = specialization,color = (gender))) +
stat_pointintervalh(.width = c(.8,.5))+ labs(y = "Specialization")+ labs(x = "Stature (z-score), posterior predictions")

h.sex.s<-bball %>%
data_grid(age_group,specialization,gender, phv_cat,state) %>%
add_predicted_draws(m4) %>%
ggplot(aes(x = .prediction, y = gender)) +
```

```
stat_pointintervalh(.width = c(.8,.5))+ labs(y = "Gender")+ labs(x = "Stature (z-score), posterior
    predictions")

h.age.s<-bball %>%
data_grid(age_group,specialization,gender, phv_cat,state) %>%
add_predicted_draws(m4) %>%
ggplot(aes(x = .prediction, y = age_group)) +
stat_pointintervalh(.width = c(.8,.5))+ labs(y = "Age group")+ labs(x = "Stature (z-score), posterior
    predictions")

h.mat.s<-bball %>%
data_grid(age_group,specialization,gender, phv_cat,state) %>%
add_predicted_draws(m4) %>%
ggplot(aes(x = .prediction, y = phv_cat)) +
stat_pointintervalh(.width = c(.8,.5))+ labs(y = "Maturity status")+ labs(x = "Stature (z-score), posterior
    predictions")

h.state.s<-bball %>%
data_grid(age_group,specialization,gender, phv_cat,state) %>%
add_predicted_draws(m4) %>%
ggplot(aes(x = .prediction, y = state)) +
stat_pointintervalh(.width = c(.8,.5))+ labs(y = "State Basketball Federation")+ labs(x = "Stature (z-score),
    posterior predictions")

h.special.state.s<-bball %>%
data_grid(age_group,specialization,gender, phv_cat,state) %>%
add_predicted_draws(m4) %>%
ggplot(aes(x = .prediction, y = state, color=(specialization))) +
stat_pointintervalh(.width = c(.8,.5))+ labs(y = "State Basketball Federation")+ labs(x = "Stature (z-score),
    posterior predictions")

m5<-
    brm(w_s~(1|age_group)+(1|specialization)+(1|phv_cat)+(1|gender)+(1|state)+(1|specialization:ge
    nder),
data=bball,
family=gaussian, prior = c(prior(normal(0,1),class = Intercept),prior(normal(0,1),class = sd)),
    chains = 4, iter = 2000, warmup = 1000, cores = 4,
    control = list(adapt_delta = 0.99,max_treedepth = 15))

plot(m5)
pp_ckeck(m5)
summary(m5)
coef(m5)

w.special.sex.s<-bball %>%
group_by(gender) %>%
data_grid(age_group,specialization,gender, phv_cat,state) %>%
add_predicted_draws(m5) %>%
ggplot(aes(x = .prediction, y = specialization,color = (gender))) +
stat_pointintervalh(.width = c(.8,.5))+ labs(y = "Specialization")+ labs(x = "Body mass (z-score), posterior
    predictions")
```

```
w.sex.s<-bball %>%
data_grid(age_group,specialization,gender, phv_cat,state) %>%
add_predicted_draws(m5) %>%
ggplot(aes(x = .prediction, y = gender)) +
stat_pointintervalh(.width = c(.8,.5))+ labs(y = "Gender")+ labs(x = "Body mass (z-score), posterior
    predictions")

w.age.s<-bball %>%
data_grid(age_group,specialization,gender, phv_cat,state) %>%
add_predicted_draws(m5) %>%
ggplot(aes(x = .prediction, y = age_group)) +
stat_pointintervalh(.width = c(.8,.5))+ labs(y = "Age group")+ labs(x = "Body mass (z-score), posterior
    predictions")

w.mat.s<-bball %>%
data_grid(age_group,specialization,gender, phv_cat,state) %>%
add_predicted_draws(m5) %>%
ggplot(aes(x = .prediction, y = phv_cat)) +
stat_pointintervalh(.width = c(.8,.5))+ labs(y = "Maturity status")+ labs(x = "Body mass (z-score),
    posterior predictions")

w.state.s<-bball %>%
data_grid(age_group,specialization,gender, phv_cat,state) %>%
add_predicted_draws(m5) %>%
ggplot(aes(x = .prediction, y = state)) +
stat_pointintervalh(.width = c(.8,.5))+ labs(y = "State Basketball Federation")+ labs(x = "Body mass (z-
    score), posterior predictions")

w.special.state.s<-bball %>%
data_grid(age_group,specialization,gender, phv_cat,state) %>%
add_predicted_draws(m5) %>%
ggplot(aes(x = .prediction, y = state, color=(specialization))) +
stat_pointintervalh(.width = c(.8,.5))+ labs(y = "State Basketball Federation")+ labs(x = "Body mass (z-
    score), posterior predictions")

###########################################
#deliberate practice motivation

m6<-brm(excel_s~(1|age_group)+(1|specialization)+(1|phv_cat)+(1|gender)+(1|state),
    data = bball,
    family = gaussian, prior = c(prior(normal(0,1),class = Intercept),prior(normal(0,1),class = sd)),
    chains = 4, iter = 2000, warmup = 1000, cores = 4,
    control = list(adapt_delta = 0.99,max_treedepth = 15))

plot(m6)
pp_ckeck(m6)
summary(m6)
coef(m6)

excel.special.sex.s<-bball %>%
```

```
group_by(gender) %>%
data_grid(age_group,specialization,gender, phv_cat,state) %>%
add_predicted_draws(m6) %>%
ggplot(aes(x = .prediction, y = specialization,color = (gender))) +
stat_pointintervalh(.width = c(.8,.5))+ labs(y = "Specialization")+ labs(x = "Will to excel (z-score),
    posterior predictions")

excel.sex.s<-bball %>%
data_grid(age_group,specialization,gender, phv_cat,state) %>%
add_predicted_draws(m6) %>%
ggplot(aes(x = .prediction, y = gender)) +
stat_pointintervalh(.width = c(.8,.5))+ labs(y = "Gender")+ labs(x = "Will to excel (z-score), posterior
    predictions")

excel.age.s<-bball %>%
data_grid(age_group,specialization,gender, phv_cat,state) %>%
add_predicted_draws(m6) %>%
ggplot(aes(x = .prediction, y = age_group)) +
stat_pointintervalh(.width = c(.8,.5))+ labs(y = "Age group")+ labs(x = "Will to excel (z-score), posterior
    predictions")

excel.mat.s<-bball %>%
data_grid(age_group,specialization,gender, phv_cat,state) %>%
add_predicted_draws(m6) %>%
ggplot(aes(x = .prediction, y = phv_cat)) +
stat_pointintervalh(.width = c(.8,.5))+ labs(y = "Maturity status")+ labs(x = "Will to excel (z-score),
    posterior predictions")

excel.state.s<-bball %>%
data_grid(age_group,specialization,gender, phv_cat,state) %>%
add_predicted_draws(m6) %>%
ggplot(aes(x = .prediction, y = state)) +
stat_pointintervalh(.width = c(.8,.5))+ labs(y = "SState Basketball Federeation")+ labs(x = "Will to excel
    (z-score), posterior predictions")

excel.special.state.s<-bball %>%
data_grid(age_group,specialization,gender, phv_cat,state) %>%
add_predicted_draws(m6) %>%
ggplot(aes(x = .prediction, y = state, color=(specialization))) +
stat_pointintervalh(.width = c(.8,.5))+ labs(y = "State Basketball Federation")+ labs(x = "Will to excel (z-
    score), posterior predictions")

m7<-brm(compete_s~(1|age_group)+(1|specialization)+(1|phv_cat)+(1|gender)+(1|state),
    data = bball,
    family = gaussian, prior = c(prior(normal(0,1),class = Intercept),prior(normal(0,1),class = sd)),
    chains = 4, iter = 2000, warmup = 1000, cores = 4,
    control = list(adapt_delta = 0.99,max_treedepth = 15))

plot(m7)
pp_ckeck(m7)
summary(m7)
```

```
coef(m7)

compete.special.sex.s<-bball %>%
group_by(gender) %>%
data_grid(age_group,specialization,gender, phv_cat,state) %>%
add_predicted_draws(m7) %>%
ggplot(aes(x = .prediction, y = specialization,color = (gender))) +
stat_pointintervalh(.width = c(.8,.5))+ labs(y = "Specialization")+ labs(x = "Will to compete (z-score), posterior predictions")

compete.sex.s<-bball %>%
data_grid(age_group,specialization,gender, phv_cat,state) %>%
add_predicted_draws(m7) %>%
ggplot(aes(x = .prediction, y = gender)) +
stat_pointintervalh(.width = c(.8,.5))+ labs(y = "Gender")+ labs(x = "Will to compete (z-score), posterior predictions")

compete.age.s<-bball %>%
data_grid(age_group,specialization,gender, phv_cat,state) %>%
add_predicted_draws(m7) %>%
ggplot(aes(x = .prediction, y = age_group)) +
stat_pointintervalh(.width = c(.8,.5))+ labs(y = "Age group")+ labs(x = "Will to compete (z-score), posterior predictions")

compete.mat.s<-bball %>%
data_grid(age_group,specialization,gender, phv_cat,state) %>%
add_predicted_draws(m7) %>%
ggplot(aes(x = .prediction, y = phv_cat)) +
stat_pointintervalh(.width = c(.8,.5))+ labs(y = "Maturity status")+ labs(x = "Will to compete (z-score), posterior predictions")

compete.state.s<-bball %>%
data_grid(age_group,specialization,gender, phv_cat,state) %>%
add_predicted_draws(m7) %>%
ggplot(aes(x = .prediction, y = state)) +
stat_pointintervalh(.width = c(.8,.5))+ labs(y = "State Basketball Federation")+ labs(x = "Will to compete (z-score), posterior predictions")

compete.special.state.s<-bball %>%
data_grid(age_group,specialization,gender, phv_cat,state) %>%
add_predicted_draws(m7) %>%
ggplot(aes(x = .prediction, y = state, color=(specialization))) +
stat_pointintervalh(.width = c(.8,.5))+ labs(y = "State Basketball Federation")+ labs(x = "Will to compete (z-score), posterior predictions")

##########
#achievement motivation

m8<-brm(competitiveness_s~(1|age_group)+(1|specialization)+(1|phv_cat)+(1|gender)+(1|state), data = bball,
family=gaussian, prior = c(prior(normal(0,1),class = Intercept),prior(normal(0,1),class = sd)),
```

```
    chains = 4, iter = 2000, warmup = 1000, cores = 4,
    control = list(adapt_delta = 0.99,max_treedepth = 15))

plot(m8)
pp_ckeck(m8)
summary(m8)
coef(m8)

competitiveness.special.sex.s<-bball %>%
group_by(gender) %>%
data_grid(age_group,specialization,gender, phv_cat,state) %>%
add_predicted_draws(m8) %>%
ggplot(aes(x = .prediction, y = specialization,color = (gender))) +
stat_pointintervalh(.width = c(.8,.5))+ labs(y = "Specialization")+ labs(x = "Competitiveness (z-score),
    posterior predictions")

competitiveness.sex.s<-bball %>%
data_grid(age_group,specialization,gender, phv_cat,state) %>%
add_predicted_draws(m8) %>%
ggplot(aes(x = .prediction, y = gender)) +
stat_pointintervalh(.width = c(.8,.5))+ labs(y = "Gender")+ labs(x = "Competitiveness (z-score), posterior
    predictions")

competitiveness.age.s<-bball %>%
data_grid(age_group,specialization,gender, phv_cat,state) %>%
add_predicted_draws(m8) %>%
ggplot(aes(x = .prediction, y = age_group)) +
stat_pointintervalh(.width = c(.8,.5))+ labs(y = "Age group")+ labs(x = "Competitiveness (z-score),
    posterior predictions")

competitiveness.mat.s<-bball %>%
data_grid(age_group,specialization,gender, phv_cat,state) %>%
add_predicted_draws(m8) %>%
ggplot(aes(x = .prediction, y = phv_cat)) +
stat_pointintervalh(.width = c(.8,.5))+ labs(y = "Maturity status")+ labs(x = "Competitiveness (z-score),
    posterior predictions")

competitiveness.state.s<-bball %>%
data_grid(age_group,specialization,gender, phv_cat,state) %>%
add_predicted_draws(m8) %>%
ggplot(aes(x = .prediction, y = state)) +
stat_pointintervalh(.width = c(.8,.5))+ labs(y = "State Basketball Federation")+ labs(x = "Competitiveness
    (z-score), posterior predictions")

competitiveness.special.state.s<-bball %>%
data_grid(age_group,specialization,gender, phv_cat,state) %>%
add_predicted_draws(m8) %>%
ggplot(aes(x = .prediction, y = state, color=(specialization))) +
stat_pointintervalh(.width = c(.8,.5))+ labs(y = "State Basketball Federation")+ labs(x = "Competitiveness
    (z-score), posterior predictions")
```

```
m9<-brm(work_s~(1|age_group)+(1|specialization)+(1|phv_cat)+(1|gender)+(1|state),
data=bball,
    family = gaussian, prior = c(prior(normal(0,1),class = Intercept),prior(normal(0,1),class = sd)),
    chains = 4, iter = 2000, warmup = 1000, cores = 4,
    control = list(adapt_delta = 0.99,max_treedepth = 15))

plot(m9)
pp_ckeck(m9)
summary(m9)
coef(m9)

work.special.sex.s<-bball %>%
group_by(gender) %>%
data_grid(age_group,specialization,gender, phv_cat,state) %>%
add_predicted_draws(m9) %>%
ggplot(aes(x = .prediction, y = specialization,color = (gender))) +
stat_pointintervalh(.width = c(.8,.5))+ labs(y = "Specialization")+ labs(x = "Work (z-score), posterior
    predictions")

work.sex.s<-bball %>%
data_grid(age_group,specialization,gender, phv_cat,state) %>%
add_predicted_draws(m9) %>%
ggplot(aes(x = .prediction, y = gender)) +
stat_pointintervalh(.width = c(.8,.5))+ labs(y = "Gender")+ labs(x = "Work (z-score), posterior
    predictions")

work.age.s<-bball %>%
data_grid(age_group,specialization,gender, phv_cat,state) %>%
add_predicted_draws(m9) %>%
ggplot(aes(x = .prediction, y = age_group)) +
stat_pointintervalh(.width = c(.8,.5))+ labs(y = "Age group")+ labs(x = "Work (z-score), posterior
    predictions")

work.mat.s<-bball %>%
data_grid(age_group,specialization,gender, phv_cat,state) %>%
add_predicted_draws(m9) %>%
ggplot(aes(x = .prediction, y = phv_cat)) +
stat_pointintervalh(.width = c(.8,.5))+ labs(y = "Maturity status")+ labs(x = "Work (z-score), posterior
    predictions")

work.state.s<-bball %>%
data_grid(age_group,specialization,gender, phv_cat,state) %>%
add_predicted_draws(m9) %>%
ggplot(aes(x = .prediction, y = state)) +
stat_pointintervalh(.width = c(.8,.5))+ labs(y = "State Basketball Federation")+ labs(x = "Work (z-score),
    posterior predictions")

work.special.state.s<-bball %>%
data_grid(age_group,specialization,gender, phv_cat,state) %>%
add_predicted_draws(m9) %>%
ggplot(aes(x = .prediction, y = state, color = (specialization))) +
```

```
stat_pointintervalh(.width = c(.8,.5))+ labs(y = "State Basketball Federation")+ labs(x = "Work (z-score),
    posterior predictions")

m10<-brm(mastery_s~(1|age_group)+(1|specialization)+(1|phv_cat)+(1|gender)+(1|state),
    data = bball,
    family = gaussian,
    prior = c(prior(normal(0,1),class = Intercept),prior(normal(0,1),class = sd)),
    chains = 4, iter = 2000, warmup = 1000, cores = 4,
    control = list(adapt_delta = 0.99,max_treedepth = 15))

plot(m10)
pp_ckeck(m10)
summary(m10)
coef(m10)

mastery.special.sex.s<-bball %>%
group_by(gender) %>%
data_grid(age_group,specialization,gender, phv_cat,state) %>%
add_predicted_draws(m10) %>%
ggplot(aes(x = .prediction, y = specialization,color = (gender))) +
stat_pointintervalh(.width = c(.8,.5))+ labs(y = "Specialization")+ labs(x = "Mastery (z-score), posterior
    predictions")

mastery.sex.s<-bball %>%
data_grid(age_group,specialization,gender, phv_cat,state) %>%
add_predicted_draws(m10) %>%
ggplot(aes(x = .prediction, y = gender)) +
stat_pointintervalh(.width = c(.8,.5))+ labs(y = "Gender")+ labs(x = "Mastery (z-score), posterior
    predictions")

mastery.age.s<-bball %>%
data_grid(age_group,specialization,gender, phv_cat,state) %>%
add_predicted_draws(m10) %>%
ggplot(aes(x = .prediction, y = age_group)) +
stat_pointintervalh(.width = c(.8,.5))+ labs(y = "Age group")+ labs(x = "Mastery (z-score), posterior
    predictions")

mastery.mat.s<-bball %>%
data_grid(age_group,specialization,gender, phv_cat,state) %>%
add_predicted_draws(m10) %>%
ggplot(aes(x = .prediction, y = phv_cat)) +
stat_pointintervalh(.width = c(.8,.5))+ labs(y = "Maturity status")+ labs(x = "Mastery (z-score), posterior
    predictions")

mastery.state.s<-bball %>%
data_grid(age_group,specialization,gender, phv_cat,state) %>%
add_predicted_draws(m10) %>%
ggplot(aes(x = .prediction, y = state)) +
stat_pointintervalh(.width = c(.8,.5))+ labs(y = "State Basketball Federation")+ labs(x = "Mastery (z-
    score), posterior predictions")
```

```
mastery.special.state.s<-bball %>%
data_grid(age_group,specialization,gender, phv_cat,state) %>%
add_predicted_draws(m10) %>%
ggplot(aes(x = .prediction, y = state, color=(specialization))) +
stat_pointintervalh(.width = c(.8,.5))+ labs(y = "State Basketball Federation")+ labs(x = "Mastery (z-
    score), posterior predictions")

##############################
#enjoyment


m11<-brm(self_ref_comp_s~(1|age_group)+(1|specialization)+(1|phv_cat)+(1|gender)+(1|state),
    data=bball,
family=gaussian, prior=c(prior(normal(0,1),class=Intercept),prior(normal(0,1),class=sd)),
    chains = 4, iter = 2000, warmup = 1000, cores = 4,
    control = list(adapt_delta=0.99,max_treedepth=15))

plot(m11)
pp_ckeck(m11)
summary(m11)
coef(m11)

self_ref_comp.special.sex.s<-bball %>%
group_by(gender) %>%
data_grid(age_group,specialization,gender, phv_cat,state) %>%
add_predicted_draws(m11) %>%
ggplot(aes(x = .prediction, y = specialization,color = (gender))) +
stat_pointintervalh(.width = c(.8,.5))+ labs(y = "Specialization")+ labs(x = "Self-reference competences (z-
    score), posterior predictions")

self_ref_comp.sex.s<-bball %>%
data_grid(age_group,specialization,gender, phv_cat,state) %>%
add_predicted_draws(m11) %>%
ggplot(aes(x = .prediction, y = gender)) +
stat_pointintervalh(.width = c(.8,.5))+ labs(y = "Gender")+ labs(x = "Self-reference competences (z-score),
    posterior predictions")

self_ref_comp.age.s<-bball %>%
data_grid(age_group,specialization,gender, phv_cat,state) %>%
add_predicted_draws(m11) %>%
ggplot(aes(x = .prediction, y = age_group)) +
stat_pointintervalh(.width = c(.8,.5))+ labs(y = "Age group")+ labs(x = "Self-reference competences (z-
    score), posterior predictions")

self_ref_comp.mat.s<-bball %>%
data_grid(age_group,specialization,gender, phv_cat,state) %>%
add_predicted_draws(m11) %>%
ggplot(aes(x = .prediction, y = phv_cat)) +
stat_pointintervalh(.width = c(.8,.5))+ labs(y = "Maturity status")+ labs(x = "Self-reference competences
    (z-score), posterior predictions")
```

```
self_ref_comp.state.s<-bball %>%
data_grid(age_group,specialization,gender, phv_cat,state) %>%
add_predicted_draws(m11) %>%
ggplot(aes(x = .prediction, y = state)) +
stat_pointintervalh(.width = c(.8,.5))+ labs(y = "State Basketball Federation")+ labs(x = "Self-reference
    competences (z-score), posterior predictions")

self_ref_comp.special.state.s<-bball %>%
data_grid(age_group,specialization,gender, phv_cat,state) %>%
add_predicted_draws(m11) %>%
ggplot(aes(x = .prediction, y = state)) +
stat_pointintervalh(.width = c(.8,.5))+ labs(y = "State Basketball Federation")+ labs(x = "Self-reference
    competences (z-score), posterior predictions")

m12<-brm(other_ref_comp_s~(1|age_group)+(1|specialization)+(1|phv_cat)+(1|gender)+(1|state),
data = bball,
family=gaussian,
prior=c(prior(normal(0,1),class = Intercept),prior(normal(0,1),class = sd)),
chains = 4, iter = 2000, warmup = 1000, cores = 4,
control = list(adapt_delta = 0.99,max_treedepth = 15))

plot(m12)
pp_ckeck(m12)
summary(m12)
coef(m12)

other_ref_comp.special.sex.s<-bball %>%
group_by(gender) %>%
data_grid(age_group,specialization,gender, phv_cat,state) %>%
add_predicted_draws(m12) %>%
ggplot(aes(x = .prediction, y = specialization,color = (gender))) +
stat_pointintervalh(.width = c(.8,.5))+ labs(y = "Specialization")+ labs(x = "Other-reference competences
    (z-score), posterior predictions")

other_ref_comp.sex.s<-bball %>%
data_grid(age_group,specialization,gender, phv_cat,state) %>%
add_predicted_draws(m12) %>%
ggplot(aes(x = .prediction, y = gender)) +
stat_pointintervalh(.width = c(.8,.5))+ labs(y = "Gender")+ labs(x = "Other-reference competences (z-
    score), posterior predictions")

other_ref_comp.age.s<-bball %>%
data_grid(age_group,specialization,gender, phv_cat,state) %>%
add_predicted_draws(m12) %>%
ggplot(aes(x = .prediction, y = age_group)) +
stat_pointintervalh(.width = c(.8,.5))+ labs(y = "Age group")+ labs(x = "Other-reference competences (z-
    score), posterior predictions")

other_ref_comp.mat.s<-bball %>%
data_grid(age_group,specialization,gender, phv_cat,state) %>%
add_predicted_draws(m12) %>%
```

```
ggplot(aes(x = .prediction, y = phv_cat)) +
stat_pointintervalh(.width = c(.8,.5))+ labs(y = "Maturity status")+ labs(x = "Other-reference
    competences (z-score), posterior predictions")

other_ref_comp.state.s<-bball %>%
data_grid(age_group,specialization,gender, phv_cat,state) %>%
add_predicted_draws(m12) %>%
ggplot(aes(x = .prediction, y = state)) +
stat_pointintervalh(.width = c(.8,.5))+ labs(y = "State Basketball Federation")+ labs(x = "Other-reference
    competences (z-score), posterior predictions")

other_ref_comp.special.state.s<-bball %>%
group_by(specialization) %>%
data_grid(age_group,specialization,gender, phv_cat,state) %>%
add_predicted_draws(m12) %>%
ggplot(aes(x = .prediction, y = state,color = (specialization))) +
stat_pointintervalh(.width = c(.8,.5))+ labs(y = "State Basketball Federation")+ labs(x = "Other-reference
    competence (z-score), posterior predictions")

m13<-brm(effort_exp_s~(1|age_group)+(1|specialization)+(1|phv_cat)+(1|gender)+(1|state),
data = bball,
family = gaussian, prior = c(prior(normal(0,1),class = Intercept),prior(normal(0,1),class = sd)),
    chains = 4, iter = 2000, warmup = 1000, cores = 4,
control = list(adapt_delta = 0.99,max_treedepth = 15))

plot(m13)
pp_ckeck(m13)
summary(m13)
coef(m13)

effort_exp.special.sex.s<-bball %>%
group_by(gender) %>%
data_grid(age_group,specialization,gender, phv_cat,state) %>%
add_predicted_draws(m13)%>%
ggplot(aes(x = .prediction, y = specialization,color = (gender))) +
stat_pointintervalh(.width = c(.8,.5))+ labs(y = "Specialization")+ labs(x = "Effort expenditure (z-score),
posterior predictions")

effort_exp.sex.s<-bball %>%
data_grid(age_group,specialization,gender, phv_cat,state) %>%
add_predicted_draws(m13)%>%
ggplot(aes(x = .prediction, y = gender)) +
stat_pointintervalh(.width = c(.8,.5))+ labs(y = "Gender")+ labs(x = "Effort expenditure (z-score),
posterior predictions")

effort_exp.age.s<-bball %>%
data_grid(age_group,specialization,gender, phv_cat,state) %>%
add_predicted_draws(m13)%>%
ggplot(aes(x = .prediction, y = age_group)) +
stat_pointintervalh(.width = c(.8,.5))+ labs(y = "Age group")+ labs(x = "Effort expenditure (z-score),
posterior predictions")
```

```
effort_exp.mat.s<-bball %>%
data_grid(age_group,specialization,gender, phv_cat,state) %>%
add_predicted_draws(m13)%>%
ggplot(aes(x = .prediction, y = phv_cat)) +
stat_pointintervalh(.width = c(.8,.5))+ labs(y = "Maturity status")+ labs(x = "Effort expenditure (z-score), posterior predictions")

effort_exp.state.s<-bball %>%
data_grid(age_group,specialization,gender, phv_cat,state) %>%
add_predicted_draws(m13)%>%
ggplot(aes(x = .prediction, y = state)) +
stat_pointintervalh(.width = c(.8,.5))+ labs(y = "State Basketball Federation")+ labs(x = "Effort expenditure (z-score), posterior predictions")

effort_exp.special.state.s<-bball %>%
group_by(specialization) %>%
data_grid(age_group,specialization,gender, phv_cat,state) %>%
add_predicted_draws(m13) %>%
ggplot(aes(x = .prediction, y = state,color = (specialization))) +
stat_pointintervalh(.width = c(.8,.5))+ labs(y = "State Basketball Federation")+ labs(x = "Effort expenditure (z-score), posterior predictions")

m14<- brm(pos_parent_involv_s~(1|age_group)+(1|specialization)+(1|phv_cat)+(1|gender)+(1|state),
    data=bball,
family=gaussian,
prior=c(prior(normal(0,1),class = Intercept),prior(normal(0,1),class = sd)),
chains = 4, iter = 2000, warmup = 1000, cores = 4,
control = list(adapt_delta = 0.99,max_treedepth = 15))

plot(m14)
pp_ckeck(m14)
summary(m14)
coef(m14)

pos_parent_involv.special.sex.s<-bball %>%
group_by(gender) %>%
data_grid(age_group,specialization,gender, phv_cat,state) %>%
add_predicted_draws(m14)%>%
ggplot(aes(x = .prediction, y = specialization,color = (gender))) +
stat_pointintervalh(.width = c(.8,.5))+ labs(y = "Specialization")+ labs(x = "Positive parental involvement (z-score), posterior predictions")

pos_parent_involv.sex.s<-bball %>%
data_grid(age_group,specialization,gender, phv_cat,state) %>%
add_predicted_draws(m14) %>%
ggplot(aes(x = .prediction, y = gender)) +
stat_pointintervalh(.width = c(.8,.5))+ labs(y = "Gender")+ labs(x = "Positive parental involvement (z-score), posterior predictions")

pos_parent_involv.age.s<-bball %>%
```

```
data_grid(age_group,specialization,gender, phv_cat,state) %>%
add_predicted_draws(m14) %>%
ggplot(aes(x = .prediction, y = age_group)) +
stat_pointintervalh(.width = c(.8,.5))+ labs(y= "Age group")+ labs(x= "Positive parental involvement (z-score), posterior predictions")

pos_parent_involv.mat.s<-bball %>%
data_grid(age_group,specialization,gender, phv_cat,state) %>%
add_predicted_draws(m14) %>%
ggplot(aes(x = .prediction, y = phv_cat)) +
stat_pointintervalh(.width = c(.8,.5))+ labs(y= "Maturity status")+ labs(x= "Positive parental involvement (z-score), posterior predictions")

pos_parent_involv.state.s<-bball %>%
 data_grid(age_group,specialization,gender, phv_cat,state) %>%
add_predicted_draws(m14) %>%
ggplot(aes(x = .prediction, y = state)) +
stat_pointintervalh(.width = c(.8,.5))+ labs(y = "State Basketball Federation")+ labs(x = "Positive parental involvement (z-score), posterior predictions")

pos_parent_involv.special.state.s<-bball %>%
group_by(specialization) %>%
data_grid(age_group,specialization,gender, phv_cat,state) %>%
add_predicted_draws(m14) %>%
ggplot(aes(x = .prediction, y = state,color = (specialization))) +
stat_pointintervalh(.width = c(.8,.5))+ labs(y = "State Basketball Federation")+ labs(x = "Positive parental involvement (z-score), posterior predictions")

m15<-brm(affil_peer_s~(1|age_group)+(1|specialization)+(1|phv_cat)+(1|gender)+(1|state),
data=bball,
family=gaussian,
    prior=c(prior(normal(0,5),class = Intercept),prior(normal(0,2.5),class = sd)),
chains = 4, iter = 2000, warmup = 1000, cores = 4,
control = list(adapt_delta=0.99,max_treedepth = 15))

plot(m15)
pp_ckeck(m15)
summary(m15)
coef(m15)


affil_peer.special.sex.s<-bball %>%
group_by(gender) %>%
data_grid(age_group,specialization,gender, phv_cat,state) %>%
add_predicted_draws(m15) %>%
ggplot(aes(x = .prediction, y = specialization,color = (gender))) +
stat_pointintervalh(.width = c(.8,.5))+ labs(y = "Specialization")+ labs(x = "Affiliation with peers (z-score), posterior predictions")

affil_peer.sex.s<-bball %>%
data_grid(age_group,specialization,gender, phv_cat,state) %>%
```

```
add_predicted_draws(m15) %>%
ggplot(aes(x = .prediction, y = gender)) +
stat_pointintervalh(.width = c(.8,.5))+ labs(y = "Gender")+ labs(x = "Affiliation with peers (z-score), posterior predictions")

affil_peer.age.s<-bball %>%
data_grid(age_group,specialization,gender, phv_cat,state) %>%
add_predicted_draws(m15) %>%
ggplot(aes(x = .prediction, y = age_group)) +
stat_pointintervalh(.width = c(.8,.5))+ labs(y = "Age group")+ labs(x = "Affiliation with peers (z-score), posterior predictions")

affil_peer.mat.s<-bball %>%
data_grid(age_group,specialization,gender, phv_cat,state) %>%
add_predicted_draws(m15) %>%
ggplot(aes(x = .prediction, y = phv_cat)) +
stat_pointintervalh(.width = c(.8,.5))+ labs(y= "Maturity status")+ labs(x= "Affiliation with peers (z-score), posterior predictions")

affil_peer.state.s<-bball %>%
data_grid(age_group,specialization,gender, phv_cat,state) %>%
add_predicted_draws(m15) %>%
ggplot(aes(x = .prediction, y = state)) +
stat_pointintervalh(.width = c(.8,.5))+ labs(y= "State Basketball Federation")+ labs(x= "Affiliation with peers (z-score), posterior predictions")

affil_peer.special.state.s<-bball %>%
group_by(specialization) %>%
data_grid(age_group,specialization,gender, phv_cat,state) %>%
add_predicted_draws(m15) %>%
ggplot(aes(x = .prediction, y = state,color = (specialization))) +
stat_pointintervalh(.width = c(.8,.5))+ labs(y = "State Basketball Federation")+ labs(x = "Affiliation with peers (z-score), posterior predictions")
```
